# Multiple mutations in the *para*-sodium channel gene are associated with pyrethroid resistance in *Rhipicephalus microplus* from the United States and Mexico

**DOI:** 10.1186/s13071-014-0456-z

**Published:** 2014-10-01

**Authors:** Nathan E Stone, Pia U Olafson, Ronald B Davey, Greta Buckmeier, Deanna Bodine, Lindsay C Sidak-Loftis, John R Giles, Roberta Duhaime, Robert J Miller, Juan Mosqueda, Glen A Scoles, David M Wagner, Joseph D Busch

**Affiliations:** Center for Microbial Genetics and Genomics, Northern Arizona University, 1298 S Knoles Drive, Flagstaff, AZ 86011-4073 USA; USDA, ARS, Knipling-Bushland United States Livestock Insects Research Laboratory, 2700 Fredericksburg Rd, Kerrville, TX 78028 USA; USDA, ARS, SPA, Cattle Fever Tick Research Laboratory, Moore Air Base, Building 6419, 22675 N. Moorefield Road, Edinburg, TX 78541 USA; Environmental Futures Research Institute, Griffith University, Brisbane, 4111 Australia; USDA-APHIS, Veterinary Services, 903 San Jacinto Blvd., Room 220, Austin, TX 78701 USA; Universidad Autónoma de Querétaro, Av. de las Ciencias S/N, Juriquilla, Queretaro, C.P 76230 Mexico; USDA, ARS, Animal Disease Research Unit, 3003 ADBF, Washington State University, Pullman, WA 99164 USA

**Keywords:** Bovine babesiosis, *Para*-sodium channel gene, Pyrethroid resistance, *Rhipicephalus microplus*, *Rhipicephalus annulatus*, *Super-kdr*

## Abstract

**Background:**

Acaricide resistant *Rhipicephalus microplus* populations have become a major problem for many cattle producing areas of the world. Pyrethroid resistance in arthropods is typically associated with mutations in domains I, II, III, and IV of voltage-gated sodium channel genes. In *R. microplus*, known resistance mutations include a domain II change (C190A) in populations from Australia, Africa, and South America and a domain III mutation (T2134A) that only occurs in Mexico and the U.S.

**Methods:**

We investigated pyrethroid resistance in cattle fever ticks from Texas and Mexico by estimating resistance levels in field-collected ticks using larval packet discriminating dose (DD) assays and identifying single nucleotide polymorphisms (SNPs) in the *para*-sodium channel gene that associated with resistance. We then developed qPCR assays for three SNPs and screened a larger set of 1,488 *R. microplus* ticks, representing 77 field collections and four laboratory strains, for SNP frequency.

**Results:**

We detected resistance SNPs in 21 of 68 U.S. field collections and six of nine Mexico field collections. We expected to identify the domain III SNP (T2134A) at a high frequency; however, we only found it in three U.S. collections. A much more common SNP in the U.S. (detected in 19 of 21 field collections) was the C190A domain II mutation, which has never before been reported from North America. We also discovered a novel domain II SNP (T170C) in ten U.S. and two Mexico field collections. The T170C transition mutation has previously been associated with extreme levels of resistance (*super*-knockdown resistance) in insects. We found a significant correlation (*r* = 0.81) between the proportion of individuals in field collections that carried any two resistance SNPs and the percent survivorship of F1 larvae from these collections in DD assays. This relationship is accurately predicted by a simple linear regression model (R^2^ = 0.6635).

**Conclusions:**

These findings demonstrate that multiple mutations in the *para*-sodium channel gene independently associate with pyrethroid resistance in *R. microplus* ticks, which is likely a consequence of human-induced selection.

**Electronic supplementary material:**

The online version of this article (doi:10.1186/s13071-014-0456-z) contains supplementary material, which is available to authorized users.

## Background

Chemical control of arthropod pests is of great importance to agricultural production and human health. Unfortunately, selection pressure from insecticides can rapidly lead to the development of resistant populations, such as the widely distributed resistance to DDT developed by flies and mosquitoes during the 20th century [1,2]. Also commonly observed is resistance to pyrethroids, which has arisen in multiple lineages of insects and ticks [3-5] and represents a significant pest control problem worldwide. Pyrethroid resistance is based on multiple mutations in voltage-gated sodium channels that result in “knockdown resistance” (*kdr*) that prevents the channel deactivation activity caused by pyrethroids [6-8]. In susceptible individuals, pyrethroids maintain the open configuration of voltage-gated sodium channels, resulting in paralysis. Some mutations are held in common across a wide phylogenetic range [6], whereas others may be specific to certain lineages, such as the domain I V421M mutation in the tobacco budworm (*Heliothis virscens*) [9]. The mutations that underlie knockdown resistance (hereafter resistance SNPs) have been the catalyst for extensive research efforts in recent decades, and are among the most studied of any resistance mechanism [8].

The southern cattle tick (*Rhipicephalus microplus*) has remained a significant burden to the global cattle industry for more than a century. This highly invasive ectoparasite has become established in many tropical and subtropical regions throughout the world, and is the biological vector (along with the closely related cattle tick; *R. annulatus*) of *Babesia bovis* and *B. bigemina*, the protozoan parasites that cause bovine babesiosis (cattle fever) and *Anaplasma marginale*, the causative agent of anaplasmosis. Biological transmission of *B. bovis* and *B. bigemina* can only occur through a tick vector [10,11]; therefore, the disease can be prevented by eradicating both *Rhipicephalus* species (collectively referred to as cattle fever ticks). This insight led to the establishment of the National Cattle Fever Tick Eradication Program (CFTEP) in the U.S. during the early 1900s. This program was very successful and led to the eradication of both tick species from the U.S. by 1960. Had eradication not been successful, the USDA-APHIS estimates that the economic losses caused by this tick-vector system would be approximately $1 billion annually [12].

**Figure 1:**
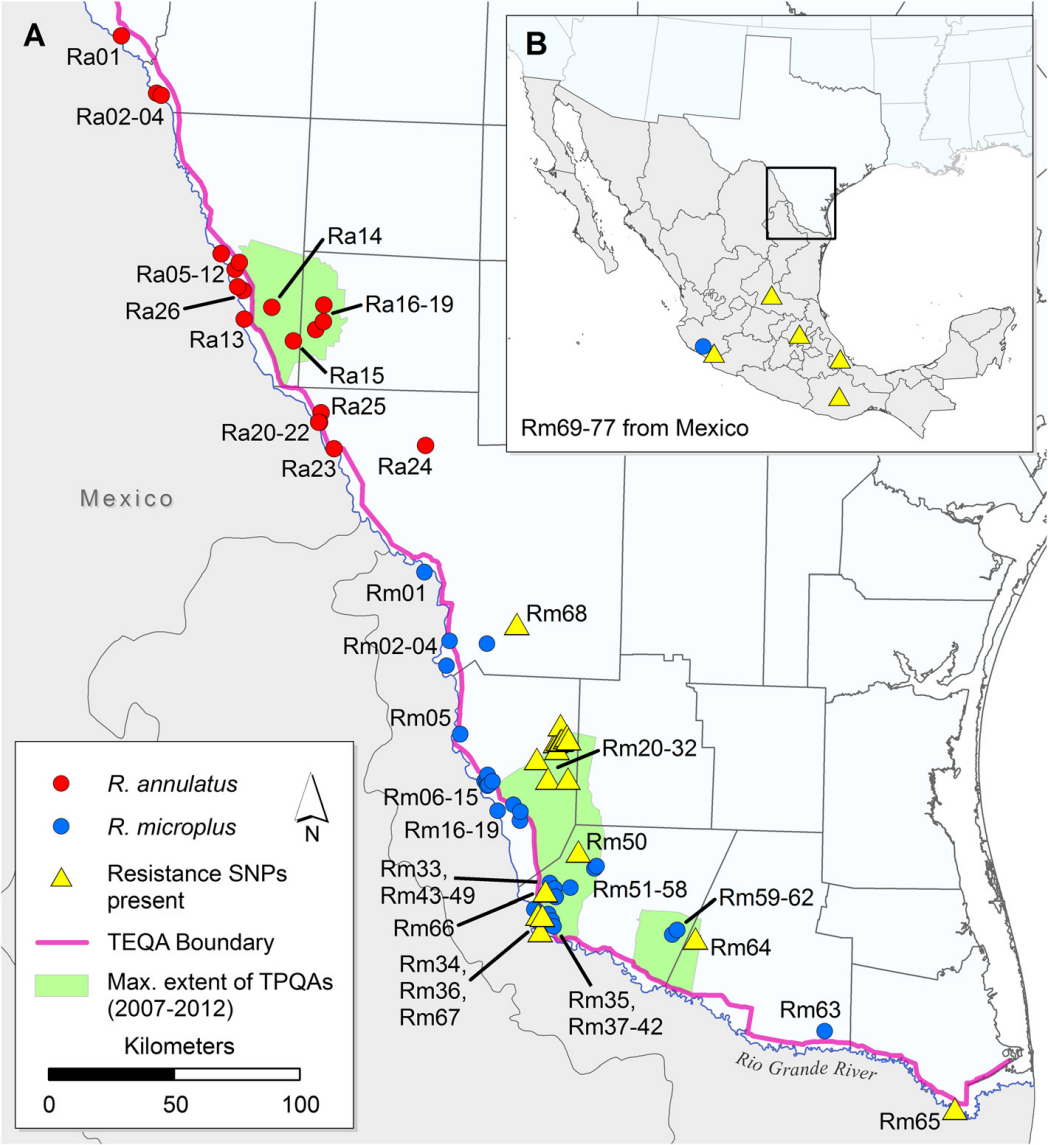
**Sampling locations for all** ***Rhipicephalus microplus*** **and** ***R. annulatus*** **collections screened for resistance SNPs in this study.** Panel **A)** All *R. microplus* collections exhibiting at least one resistance SNP (detected via quantitative PCR or Sanger sequencing) are marked with a yellow triangle. No resistance SNPs were detected in any *R. annulatus* collections (Additional file 1: Table S1). The pink line delineates the northern boundary of the permanent tick eradication quarantine area (TEQA) that is maintained along the international border of Texas and Mexico, while the green polygons represent the maximum extent of three temporary preventative quarantine areas (TPQAs) that were implemented from 2007–2012. Panel **B)** Mexico collections (Rm 69–77) are represented at the state level in the inset map. Only three collection sites in Mexico (Rm69, 72, and73) carried fully susceptible genotypes, shown as a blue circle in the state of Colima (see Additional file 1: Table S1 for details).

The reintroduction of *Babesia* and cattle fever ticks into the U.S. is a constant threat as this disease-vector system remains endemic in Mexico. Reintroduction is only prevented by a narrow quarantine zone that runs ~800 km along the Rio Grande border with Mexico; it is maintained by the U.S. Department of Agriculture-Animal Plant Health Inspection Service-Veterinary Services division (USDA-APHIS-VS) (Figure 1). All cattle that are imported into the U.S. from Mexico (>1 million annually) [13] must enter through one of four Texas-controlled ports of entry within the tick eradication quarantine area (TEQA) and determined to be tick free. This involves physically inspecting each animal and then dipping them in the organophosphate acaricide coumaphos. Following these procedures the cattle can then be transported beyond the TEQA to feedlots or slaughter facilities but are not allowed to remain within it. Despite rigorous efforts to prevent tick movements into southern Texas, new infestations are being discovered both in the TEQA and beyond it in temporary preventative quarantine areas (TPQAs) (Figure 1) [14,15].

Maintaining tick eradication in Texas relies almost exclusively on acaricide treatment but widespread acaricide resistance in Mexico threatens the success of the eradication program, especially when it occurs in states that border the U.S. [16-18]. This is highlighted by an increasing number of infestations in the TEQA and beyond that have been found in recent years to be resistant [19,20]. Nearly all resistant infestations discovered within the U.S. are associated with pyrethroid acaricides, however, resistance to multiple acaricide classes (including coumaphos) has been described in the U.S. [14,19] and Mexico [21,22]. Pyrethroid resistance was first discovered in Mexico in 1994, shortly after the introduction of pyrethroids into the Mexican cattle market in the 1980s [23]. Since coumaphos is the only acaricide used in the TEQA dipping vats, treating infestations of coumaphos-resistant ticks requires the use of a different acaricide class (such as pyrethroids). Therefore, the ability to rapidly detect acaricide resistance mutations (of any class) and predict resistance levels in Texas is important for preventing the spread of resistant tick populations. In addition, understanding the evolution of resistance to pyrethroids and other acaricide classes, as well as uncovering the diversity of resistance mechanisms that exist, are clearly important for pest management issues in all parts of the world.

Pyrethroid resistance in arthropods is typically associated with non-synonymous mutations in domains I, II, III, and IV of voltage-gated sodium channel genes [6,8]. In *R. microplus* the specific voltage-gated sodium channel associated with pyrethroid resistance is the *para*-sodium channel, and resistance mechanisms in domains II and III of this gene have been described previously in *R. microplus* ticks. A domain II mutation (C190A) occurs in southern cattle tick populations on three continents (Australia, Africa, and South America) [24-26], whereas the domain III SNP (T2134A) has only been reported in ticks from Mexico and Texas [19,26,27]. Although resistance in *R. microplus* populations from Mexico has been attributed largely to the domain III SNP T2134A [17,27,28], several authors have suggested the likelihood of additional resistance mechanisms in the *para*-sodium channel gene because genotype frequencies from screened populations did not account for the level of phenotypic resistance observed [17,24,27]. Thus, further evaluation of the mechanisms underlying pyrethroid resistance in *R. microplus* ticks is important.

Here we describe the presence of not just one but three *para*-sodium channel SNP mutations in field-collected tick samples from the U.S. and Mexico. We detected the two SNPs (C190A & T2134A) previously described from resistant tick populations [25,27], but also discovered a novel SNP (M918T/T170C) known to account for *super-*knockdown resistance (*super-kdr*) in insects [29-31] that has never been reported in ticks. Our data suggest that these multiple mutations in *R. microplus* act in concert to increase pyrethroid resistance, particularly when individuals possess a combination of any two resistance SNPs at any of the three loci. Our study provides a straightforward method to predict the level of pyrethroid resistance in *R. microplus* populations, which has significance for all major cattle-producing regions of the world that control cattle fever ticks with acaricides.

## Methods

### Tick collections and acaricide testing

We utilized *Rhipicephalus microplus* ticks that were representative of the genetic and phenotypic diversity within the TEQA, TPQAs, and Mexico. In total, 1488 *R. microplus* ticks obtained from three separate sources were used for this study: 1) 68 USDA-APHIS field collections from southern Texas, 2) nine Mexico field collections, and 3) four USDA-ARS laboratory strains (Additional file 1: Table S1)*.* A tick collection is defined as a sample of ticks collected from a single property within a thirty day time window, as previously described by Busch *et al.* [14]. The most important hosts for *R. microplus* in southern Texas are cattle (mixed *Bos taurus/B. indicus* breeds) and white-tailed deer (*Odocoileus virginianus*). The USDA-APHIS field collections were made from both hosts, but the other sampling sources (Mexico field collections and ARS laboratory strains) were from cattle only. All U.S. field collections (*n* = 68) were sampled by APHIS personnel at 51 properties in southern Texas, as described by Busch *et al*. [14]. Our study utilized excess field ticks that were not needed for larval packet discriminating dose (DD) assays (see section below on acaricide testing) and had been stored frozen in an archive maintained by the USDA-ARS in Kerrville, TX. We used 1,247 ticks from 2005–2010 described by Busch *et al.* [14], an additional 27 ticks from collection Rm50, four recent (2010–2011) APHIS collections (Rm64-Rm67, *n* = 82) known to be resistant to permethrin (the specific pyrethroid used in USDA-ARS larval packet DD assays), and a single tick (Rm68, or NVSL_T620) collected in Webb County and archived by the National Veterinary Sciences Laboratory in Ames, IA. Mexican field collections were sampled by the Universidad Autónoma de Querétaro at nine properties throughout central Mexico in 2012 (*n* = 31).

Individual larval samples from four laboratory strains maintained by the USDA-ARS Cattle Fever Tick Research Laboratory (CFTRL; Edinburg, TX) were genetically analyzed using three resistance SNP assays (see Molecular methods). These strains were identified by DD assays as exhibiting varying levels of permethrin resistance: B&H Ranch (*n* = 10 larvae; F_6_ generation) [19], Corrales (*n* = 30 larvae; F_4_ generation), San Felipe (*n* = 30 larvae; F_44_ generation), and Santa Luiza (*n* = 30 larvae; F_20_ generation) [24]. These same laboratory strains have been analyzed extensively in previous studies that examined pyrethroid resistance [19,24,27,28,32-34].

Resistance to pyrethroids is not commonly observed in *Rhipicephalus annulatus* ticks, yet the potential for them to appear as a result of human-induced selection pressure or hybridization between *R. microplus* and *R. annulatus* is possible [35]. As such, we analyzed *R. annulatus* ticks (*n* = 434) from 26 field collections to test the effectiveness of our resistance SNP assays on this closely related species. The *Rhipicephalus annulatus* field collections were sampled by APHIS personnel at 18 properties in southern Texas [36] in the same manner as *R. microplus. R. annulatus* samples were collected from cattle, white-tailed deer, exotic red deer (*Cervus elaphus*), and domestic horses (*Equus ferus caballus*) (Additional file 1: Table S1).

Permethrin resistance in cattle fever ticks from the U.S. was initially evaluated by larval packet DD assays. Briefly, semi to fully engorged wild adult female ticks that were collected from infested premises within either the TEQA or TPQAs of the CFTEP were incubated until oviposition was complete. Egg masses from multiple females were then thoroughly mixed and a random sampling of hundreds of 14 day old F_1_ larvae were assayed using the DD test [37]. The permethrin assay used a 0.125% concentration of active ingredient (AI) that is known to cause 99% lethality (LC_99_) in fully susceptible ticks. The 2× LC_99_ DD of 0.250% AI was also tested. Resistance is defined by the USDA as any deviation from 100% mortality as measured at either the 0.125% or 0.250% AI level. Our larval packet DD data for both species (*R. microplus* and *R. annulatus*) were generated by the CFTRL using F_1_ larvae from U.S. field collections and one laboratory strain, Corrales (F_4_). Larval packet DD assays could not be run on a subset of field collections (*n* = 38), either because sample size was too low or the field ticks were not engorged enough to lay a viable egg mass. The remaining three laboratory strains (B&H Ranch, Santa Luiza, and San Felipe) were subject to full bioassays to determine LC_50_ and LC_99_, which uses a different range of % AI that is not directly comparable to the DD assays. As such, larval packet DD data are not available for those strains. In addition, no larval packet DD data were collected for the Mexico field ticks.

### Molecular methods

Genomic DNA was extracted from individual adult and larval ticks from the USDA-ARS frozen tick archives and Mexico field collections (none of our samples were pooled). Extractions were performed using DNeasy kits (Qiagen, Valencia, CA, USA) according to the manufacturer’s specifications. The genomic DNA was quantified on a NanoDrop 8000 spectrophotometer (Thermo Scientific, Waltham, MA, USA) and diluted to 20 ng/μL for PCR.

We discovered resistance SNPs by querying sequences that were generated for the exons encoding domain II (*n* = 410) and domain III (*n* = 366) of the *para*-sodium channel gene from hundreds of wild *R. microplus* ticks designated as permethrin-resistant and susceptible by the larval packet DD assays. To maximize sequence coverage, the *para*-sodium channel gene mRNA sequence for *R. microplus* (putative sodium channel accession# [GenBank:AF134216.2]) was aligned to the *Ixodes scapularis* sodium channel alpha subunit gDNA sequence [https://www.vectorbase.org/ (gene: ISCW002612)] to determine the likely gene architecture for domains II and III. Exon breaks for both domains were determined and primers were designed at the exon ends.

Sequencing the exon encoding domain II was achieved using traditional Sanger sequencing methods. First, the exon was amplified using forward primer RmNaDomainIIF1 (5′TACGTGTGTTCAAGCTAGCCAA) and reverse primer RmNaDomainIIR1 (5′ACTTTCTTCGTAGTTCTTGCCAA) resulting in an amplicon length of 167 bp. PCRs were carried out in 10 μL volumes containing the following reagents (given in final concentrations): 20–40 ng of DNA template, 1× PCR buffer, 2.5 mM MgCl_2_, 0.2 mM dNTPs, 1 U Platinum® *Taq* polymerase (Invitrogen, Carlsbad, CA, USA), and 0.4 μM of each primer. PCRs were thermocycled according to the following conditions: 95°C for 10 minutes to release the polymerase antibody, followed by 40 cycles of 94°C for 60 seconds, 55°C for 30 seconds, and 72°C for 30 seconds. PCR products were then treated with ExoSAP-IT (Affymetrix, Santa Clara, CA, USA) using 1 μL of ExoSAP-IT per 7 μL of PCR product under the following conditions: 37°C for 15 minutes, followed by 80°C for 15 minutes. Treated products were then diluted 1/10 and sequenced in both directions using the same forward and reverse primers from the PCR in a BigDye® Terminator v3.1 Ready Reaction Mix (Applied Biosystems, Foster City, CA, USA). We used 10 μL volumes for sequencing reactions containing the following reagents (given in final concentrations): 5× Sequencing Buffer, 1 μL BigDye® Terminator v3.1 Ready Reaction Mix, 1 μM primer, and 5 μL diluted PCR product. The following thermocycling conditions were used: 96°C for 20 seconds, followed by 30 cycles of 96°C for 10 seconds, 50°C for 5 seconds, and 60°C for 4 minutes. As detailed below, we discovered two SNPs in domain II (T170C and C190A) that are separated by only 20 nucleotides. Therefore, we validated the allele sequences for a subset of nine heterozygous individuals by T-vector cloning and sequencing PCR amplicons. PCR products were ligated into the pGEM®-T Easy Vector System I (Promega, Madison, WI, USA), and electroporated using *E. coli* JM109 high efficiency electrocompetent cells. A total of eight colonies per individual tick were selected for PCR screening with the pUC/M13 plasmid primers, and five clones containing the correct insert size were chosen for full-coverage sequencing according to the methods described above, except the pUC/M13 primers were used.

Sequencing the domain III encoding exon proved to be more difficult and required several modifications. First, the domain III exon was amplified using forward primer RmNaDomainIIIF1 (5′AAGAGGACCAACCGGAATACG) and reverse primer RmNaDomainIIIRS2_CON (5′TCTTCTTTTGTTCATTGAAATTGT), resulting in an amplicon length of 135 bp. PCR conditions were identical to domain II conditions except for a change in annealing temperature to 53°C. Products from this first PCR were then diluted 1/1,000,000 and used as the template for a second PCR, which increased amplicon concentration and length by incorporating tails into the primers. For the second PCR we used forward tailed primer RmNaDomainIIIF3 (5′acccaactgaatagagagcAAGAGGACCAACCGGAATACG) and reverse tailed primer RmNaDomainIIIR3 (5′acgcacttgacttgtcttcTCTTCTTTTGTTCATTGAAATTGT) resulting in an amplicon length of 173 bp. The conditions of the second PCR were identical to those for the first, except the annealing temperature was increased to 65°C. After treatment with ExoSAP-IT, as described for domain II, PCR products were diluted 1/30 and sequenced in both directions using BigDye® Terminator v3.1 Ready Reaction Mix. Sequencing conditions were identical to domain II, except forward primer tail RmNaDomainIIIF3seq (5′acccaactgaatagagagc) and reverse primer tail RmNaDomainIIIR3seq (5′acgcacttgacttgtcttc) were used.

Our initial sequencing identified multiple SNPs (Table 1), three of which were correlated with permethrin resistance (see Results). Nomenclature for *para*-sodium channel gene SNPs previously published for *R. microplus* are based on nucleotide position within the mRNA sequence [GenBank:AF134216.2]; hence, we will refer to the M918T mutation as T170C. This matches the naming convention of the other SNPs (C190A and T2134A).

**Table 1:** **Multiple single nucleotide polymorphisms (SNPs) identified in the exons encoding domains II and III of the** ***para*** **-sodium channel gene in** ***Rhipicephalus microplus*** **from Texas and Mexico**

**Assay**	**Locus (Accession #)**	**Bp**	**SNP**	**AA**	**Reference**	**Notes**
**Domain II**	C190A [GenBank:KM073929]	190	C/A	Leucine/Isoleucine	Morgan et al. [25]	Three SNPs in priming site (184 bp, 189 bp, 190 bp) may cause this assay to fail
**Domain II (** ***super-kd*** **r)**	T170C [GenBank:KM073928]	170	T/C	Methionine/Threonine	Williamson et al. [29], Current Study	One SNP in priming site (148 bp) may cause this assay to fail
**Domain III**	T2134A [GenBank:KM073935]	2134	T/A	Phenylalanine/Isoleucine	He et al. [27]	One SNP in priming site (2130 bp) may cause this assay to fail
**na**	C148T [GenBank:KM073932]	148	C/T	Leucine/Phenylalanine	Current Study	Present in Rm10, Rm12, Rm13, Rm40, Rm44, Rm47, Rm48, and Rm56 (all susceptible populations)
**na**	G184C [GenBank:KM073930]	184	G/C	Glycine/Arginine	Current Study	Present in Rm70, Rm71, Rm74, Rm75, Rm76, Rm77, San Felipe, and B&H Ranch collections (occurs only in individuals from resistant populations that also carry one or two copies of the domain III T2134A resistance SNP)
**na**	C189A [GenBank:KM073931]	189	C/A	Silent	Current Study	Present in Rm64, Rm65, Rm67, and San Felipe collections (occurs with resistant genotypes only)
**na**	C190G [GenBank:KM073933]	190	C/G	Leucine/Valine	Current Study	Present in Rm77 (present in collections that also contain C190A and T2134A, unsure of its role in resistance)
**na**	C2130T [GenBank:KM073934]	2130	C/T	Silent	Current Study	Present in Rm05, Rm13, Rm36, Rm38, Rm53, Rm56, Rm65, Rm66, Rm67, Rm68, Rm75, Rm76, and Rm77 (occurs in susceptible and resistant populations)

We designed Melt-MAMA qPCR assays [38] for three SNP positions. Two assays distinguish susceptible versus resistance SNPs previously described in the exons for domain II C190A and domain III T2134A [25,27]. A third assay was designed for the putative *super*-*kdr* site (domain II T170C) observed in our study. Five other SNPs were identified from Sanger sequencing but their contribution to pyrethroid resistance in arthropods is unknown. The “Notes” column provides details on how these five SNPS may interfere with Melt-MAMA assays designed for the three resistance sites. In the case of ambiguous qPCR results, we validated all SNPs via Sanger sequencing. All SNP positions are based on annotations from the *R. microplus para*-sodium channel gene mRNA sequence (putative sodium channel accession# [GenBank:AF134216.2]).

To facilitate high-throughput identification of resistant genotypes in thousands of field ticks, we developed three rapid detection assays based on the Melt-MAMA qPCR platform [38] (Additional file 2: Table S2). We targeted SNPs T170C and C190A in domain II and T2134A in domain III. All three SNP assays were successful at detecting homozygous susceptible, homozygous resistant, and heterozygous genotypes (see Additional file 3: Figure S1) and were validated using the large subset of sequences described above. We used these three resistance SNP assays to generate multi-locus genotypes (MLGs) for all ticks. There were five additional SNPs (two synonymous and three non-synonymous) discovered in domains II and III that at times led to ambiguous genotyping calls and/or assay failures (see Table 1 for details about assay and SNP interactions). Failures and incorrect genotyping calls are easy to identify by observed deviations from expected melt profiles (see Additional file 3: Figure S1). All ambiguous results (*n* = 52) were confirmed via Sanger sequencing of the exons encoding domain II or domain III, as described above. Finally, our new genotyping methods have been adapted to a simpler agarose platform if qPCR instruments are not available (see Additional file 4: Figure S4) [38].

### Statistical analyses

We utilized data from *R. microplus* collections for three separate analyses, including: 1) frequency counts of resistance SNPs in all field and lab collections, 2) testing a correlation between the proportion of individuals in a given collection carrying any two resistance SNPs and larval packet DD percent survivorship for that collection, and 3) regression analysis of 13 U.S. field collections that contained resistant genotypes and had corresponding larval packet DD data. Although we utilized ticks from the same collection for evaluating resistance phenotype and genotype, it is important to note that a direct comparison between larval packet DD survivorship data and resistance SNP frequencies using the same individual ticks was not possible. As described above, our genetic methods utilized excess individual ticks from archived (frozen) field collections and laboratory strains, but the field-collected parental ticks and the F_1_ larvae used in the larval packet DD assays were not available for genotyping. Since the excess ticks represent a sub-sample of the parental ticks used to produce F_1_ larvae for the DD assays, we made comparisons at the population-level using larval packet DD survivorship as phenotype data and the proportion of individuals in each archived field collection carrying resistance SNPs as genotype data. Although the genotyped field ticks (mostly adults) were not used to produce F_1_ larvae for the DD assays, they were sampled from the same field collection as the parental ticks and therefore represent SNP frequencies of the same genetic population.

The first analysis allowed us to compare the resistance SNPs present in U.S. and Mexico field collections and determine their frequency. We also screened laboratory strains to check for any resistance SNPs that may have been overlooked in previous studies. Observed SNP frequencies were calculated at three loci for all field collections and laboratory strains to show the distribution and frequency of resistance SNPs (Additional file 1: Table S1).

The second analysis allowed us to determine the relationship between SNP frequency and survivorship in *R. microplus* ticks. Previous studies have noted only moderate correlations between the proportion of individuals in a population that contain at least one resistance SNP (heterozygous or homozygous resistant) at a single locus and the percent survivorship of that population in larval packet test bioassays [25,39]. Because we had MLG data from three SNP locations we were better able to investigate this association. Furthermore, we suspected this relationship might be stronger if we focused on the proportion of individuals that carried more than one resistance SNP. We calculated the observed frequencies of the six possible resistance MLGs (Table 2) that arose from the accumulation of any two resistance SNPs per individual. We also thought it was important to use field-collected ticks to initially describe this pattern because, in contrast to the laboratory collections, field-collected ticks were not experimentally challenged with permethrin. Therefore, the second dataset was limited to 13 U.S. field collections that had permethrin larval packet DD data at 0.125% and 0.250% AI. We then tested the phenotype-genotype correlation using Pearson’s correlation coefficient.

**Table 2:** **All observed SNP combinations producing six resistance multi-locus genotypes (MLGs) observed in our study**

**MLG**	**C190A**	**T170C**	**T2134A**	**U.S.**	**Mexico**	**Lab**
**1**	**RR**	**SS**	**SS**	Yes	Yes	Yes
**2**	**RS**	**RS**	**SS**	Yes	No	No
**3**	**RS**	**SS**	**RS**	Yes	Yes	Yes
**4**	**SS**	**RR**	**SS**	Yes	No	No
**5**	**SS**	**SS**	**RR**	No	Yes	Yes
**6**	**SS**	**RS**	**RS**	No	Yes	No

Each MLG has two (and only two) resistance SNPs. Specific MLGs were associated with each sampling source, although MLGs 1 and 3 were found in all three sources. Multi-locus genotype 1 was geographically widespread and the single most common MLG in our study.

We used the third dataset to predict phenotype patterns (percent larval packet DD survivorship) in F_1_ larvae using the genotypic data from the subsampling of the parental generation. A general linear regression model was fitted using SigmaPlot 2000 (Systat Software, San Jose, CA, USA) for the 13 U.S. field collections described above, using the proportion of individuals with two resistance SNPs as the independent variable and the percent survivorship at 0.250% AI as the dependent variable. In addition to the 13 collections used for the linear regression analysis, we tested data from two independent sources to evaluate the predictive ability of our simple regression model. First, we incorporated genotype and larval packet DD data from a laboratory strain, Corrales (F4 generation); and second, we added data from Morgan *et al.* that were collected from five Australian populations (four field collections and one lab strain) [25].

We also performed two population genetic analyses on our tick collections. To test for evidence of pyrethroid selection we examined Hardy-Weinberg equilibrium (HWE) at the three resistance SNP loci for collections that were not monomorphic at all three loci and had a sample size >1. We ran the HWE chi-square test on 21 field collections and one lab collection using GENALEX [40]. Additionally, we genotyped and analyzed *R. microplus* (*n* = 114) from five U.S. (Rm64-Rm68) and nine Mexico (Rm69-Rm77) field collections to determine their probability of assignment to five major genetic groups using 11 microsatellite loci and Bayesian analysis in STRUCTURE as described by Busch *et al*. [14]. These collections were acquired after the completion of these previous analyses, and in some cases, represent unique resistance MLGs that were not present in the 1247 ticks we previously described [14]. Furthermore, all of the ticks that exhibited resistant phenotypes in that previous study assigned to a single genetic group [14]. As such, we felt it was important to determine the probability of assignment for these additional samples to look for evidence of resistance SNPs in other genetic groups within the TEQA, TPQAs, and Mexico.

## Results

### Larval packet discriminating dose testing

The LC_99_ and 2× LC_99_ larval packet DD assays revealed permethrin-resistant F_1_ larvae in 12 of 77 *R. microplus* field collections (Additional file 1: Table S1). To clarify, these are 12 of the 13 field collections used to assess the correlation between phenotype and genotype (below). The 13th population (Rm28) did not demonstrate permethrin resistance according to the DD assays, even though it carried resistant genotypes (Additional file 1: Table S1). Levels of permethrin resistance were highly variable among these 12 field collections, ranging from 0.5-100% survivorship at the 0.125% AI level and 1.5-100% survivorship at the 0.250% AI level. In laboratory strains, the Corrales F_4_ larvae were highly resistant and showed 100% survivorship at both the 0.125% and 0.250% AI levels. The San Felipe, Santa Luiza, and B&H Ranch laboratory strains are also known to be resistant to permethrin, but the generations we genotyped ((F_44_), (F_20_), and (F_6_), respectively) were assessed for permethrin resistance using a different range of % AI [24,34] and, as such, are not directly comparable to the LC_99_ and 2× LC_99_ DD data. As a result, these lab strains were genotyped for resistance SNPs (below) but were not included in further analyses. All *R. annulatus* field collections exhibited 100% mortality at the 0.250% AI level, although one collection (Ra03) showed mild resistance (4.5% survivorship) at the 0.125% AI level (Additional file 1: Table S1).

### SNP discovery and genotyping

In our initial sequencing survey of the exons encoding domain II and domain III of the *para*-sodium channel gene we detected a total of eight SNPs, including three SNPs associated with resistance (at three loci) across 21 U.S. and six Mexico field collections of *R. microplus*: C190A (domain II), T170C (domain II) and T2134A (domain III) (Additional file 1: Table S1). Of the 27 field collections as a whole, 12 contained resistance SNPs from one SNP locus, 13 had representatives of two SNP loci, and two (Rm75 and Rm76 from Mexico) displayed all three mutations (Additional file 1: Table S1). Upon sequencing multiple clones from nine ticks that carried MLG 2 (see Table 2), we found that the T170C and C190A mutations resided on opposite DNA strands in all cases (see Additional file 5: Figure S2 and Additional file 6: Figure S3). In all cases, we observed a maximum of two resistance SNPs per individual. Our sequencing also revealed five additional SNPs, two synonymous and three non-synonymous (Table 1). Because their association with resistance was unclear, we chose not to investigate these SNPs further.

Our SNP typing of all available samples of *R. microplus* (*n* = 1,488) revealed that all three resistance SNPs are found in tick populations in the U.S. and Mexico, although the frequency at which they occur is highly variable between these two countries. For example, the domain III T2134A SNP is observed at a much higher frequency in Mexico than in the U.S., whereas the C190A and T170C SNPs appear to be more prevalent in the U.S. (Additional file 1: Table S1). The domain II SNP (C190A) was clearly the most common in our study. Of the 21 U.S. collections containing resistant genotypes, eight had the C190A SNP exclusively (Rm20, Rm21, Rm24, Rm25, Rm50, Rm64 [heterozygous in all four individuals], Rm66, and Rm68), nine contained both C190A and the putative *super-kdr* T170C SNP (Rm22, Rm23, Rm26, Rm27, Rm28, Rm29, Rm30, Rm31, and Rm32), two contained C190A and the domain III T2134A SNP (Rm65 and Rm67), one contained a single tick that had the putative *super-kdr* (T170C) SNP in heterozygous form (Rm34), and one contained the domain III T2134A SNP in heterozygous form at a frequency of 0.065 (Rm36) (Additional file 1: Table S1). Of the six Mexico collections that contained resistant genotypes, two contained the domain III T2134A SNP exclusively (Rm70 and Rm71), two contained both T2134A and C190A (Rm74 and Rm77), and two contained all three resistance SNPs (Rm75 and Rm76) (Additional file 1: Table S1).

As expected, all four laboratory strains contained resistant genotypes but none had the putative *super-kdr* (T170C) SNP. The Corrales and B&H Ranch laboratory strains contained the domain III T2134A SNP exclusively, as previously suspected by He *et al*. [27] and Miller *et al*. [19]. In the San Felipe strain we also found the domain III T2134A SNP at high frequency but, in addition, we detected the domain II C190A SNP in a heterozygous state at a frequency of 0.133. The C190A SNP had not been previously identified in the San Felipe strain. The Santa Luiza strain, originally procured from Brazil, was confirmed to contain the C190A SNP exclusively, as previously described by Guerrero *et al.* [32] (Additional file 1: Table S1).

All *R. annulatus* samples (*n* = 434) displayed fully susceptible MLGs across the three loci. This result corresponded to the 0.250% AI larval packet DD data (Additional file 1: Table S1) and validates the effectiveness of these assays to detect three resistance SNPs in this species. Since no resistance SNPs were observed in *R. annulatus*, no further analyses were conducted on this species.

### Population genetic analyses

All 22 *R. microplus* collections that were tested for evidence of pyrethroid selection (described above) were found to be in Hardy-Weinberg equilibrium for resistance SNP frequencies, with the exception of collections Rm50, Rm64, and Rm77. This suggests that 1) most field collections had not been sampled from a generation that was directly experiencing selection pressure from pyrethroids, and 2) mating amongst susceptible and resistant parents was random. Small sample size, rather than selection, appears to explain the deviation from HWE in collections Rm64 (*n* = 4) and Rm77 (*n* = 7). Rm50 contained a single individual (out of 56) that was homozygous resistant and thought to be a migrant tick [14]. When this tick was removed, the chi-square test was no longer significant. The San Felipe F_44_ lab strain was also in HWE. The Bayesian analysis of microsatellite genotypes did not strongly assign any of the 114 ticks from the additional five U.S. and nine Mexico collections into the four main genetic groups described by Busch *et al.* [14]. Instead, these tick collections all showed the highly admixed genetic signature typical of collections found along the Rio Grande River [14].

### Phenotype-genotype correlation and linear regression analysis

**Figure 2:**
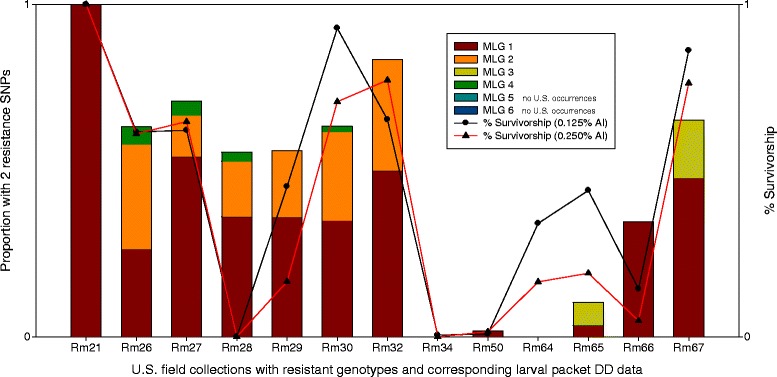
**Multi-locus genotype (MLG) proportions of 13 U.S. field collections of** ***Rhipicephalus microplus*** **with corresponding larval packet discriminating dose (DD) resistance data.** The bar for each field collection shows the total proportion of individuals with two resistance SNPs from any of three SNP loci (see Table 2); each color-coded portion represents the frequency of specific MLGs (no instances of MLG 5 or 6 occur in these 13 collections). Not shown are the proportion of individuals with a single resistance SNP (which occur in Hardy-Weinberg proportions) and those with fully susceptible MLGs. We note that no ticks were found to carry three or more resistance SNPs. The lines represent larval packet DD survivorship (%) at two permethrin AI concentrations (0.125% and 0.250%). The proportion of MLGs that carry any two resistance SNPs is highly correlated with larval packet DD survival at permethrin concentrations of 0.125% AI (*r* = 0.73, *p* = 0.0065) and 0.250% AI (*r* = 0.81, *p* = 0.0013). Collections Rm34 and Rm64 displayed a small number of resistant phenotypes in the larval packet DD assays, but neither collection had individuals with two resistance SNPs. Both had a small number of individuals with a single resistance SNP (1/42 ticks in Rm34 and 4/4 ticks in Rm64).

We observed a strong correlation between the proportion of individuals within each field collection carrying any two resistance SNPs and the percent survivorship in the larval packet DD assays (Figure 2). This pattern was significant at both the 0.125% AI (*r* = 0.73, *p* = 0.0065) and 0.250% AI (*r* = 0.81, *p* = 0.0013) concentrations. Individuals with a single resistance SNP were observed at Hardy-Weinberg proportions in these 13 collections (except Rm64, as noted above). As an *a posteriori* test, we re-ran the correlation after re-calculating SNP proportions by including all individuals with at least one resistance SNP, but this reduced the correlation coefficients (0.125% AI: *r* = 0.60, *p* = 0.041; 0.250% AI: *r* = 0.54 *p* = 0.0067). Thus, the proportion of individuals with two SNPs provided the strongest association with larval packet DD survivorship at 0.250% AI.

We observed all six possible MLGs in our study, but only MLGs 1 and 3 were shared between the U.S. and Mexico field collections and the laboratory strains (Table 2). In the 13 U.S. field collections used for phenotype-genotype correlations, we observed MLGs 1, 2, 3, and 4 but not MLGs 5 and 6. In the Mexico field collections we observed MLGs 1, 3, 5, and 6 and in the laboratory strains we observed MLGs 1, 3, and 5. In all, MLGs 1, 2, 3, and 4 (from U.S. field collections) were used to calculate Pearson’s correlation co-efficient at 0.250% AI (Figure 2). Since none of the individuals that displayed MLGs 5 and 6 had corresponding larval packet DD survivorship data, they could not be included in this analysis.

**Figure 3:**
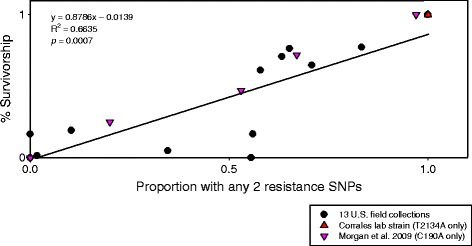
**Linear regression of 13** ***Rhipicephalus microplus*** **U.S. field collections, showing the relationship between the proportion of individuals that carry any two resistance SNPs and the larval packet discriminating dose (DD) survivorship using 0.250% AI permethrin.** This regression model was highly consistent with the data presented here from two additional sources, including ticks from Australia [25] and the Corrales laboratory strain from Mexico. Resistance in the Morgan study [25] is based on homozygous C190A domain II genotypes subjected to 0.3% cypermethrin.

To examine the predictive capabilities of the relationship between the proportion of individuals with any two resistance SNPs and the percent survivorship at the 0.250% AI level we also analyzed these data using linear regression, which yielded a significant result (R^2^ = 0.6635 *p* = 0.0007) (Figure 3). We used the 0.250% AI larval packet DD survivorship data for the linear regression because it is founded on a stronger selective pressure that should decrease the variation in permethrin survival. Additional datasets based on field and laboratory ticks from Australia and our data from the Corrales laboratory strain from Mexico are highly consistent with this linear model and support its validity (Figure 3). MLGs 1 through 5 were represented in the linear regression figure, but MLG 6 could not be included because it was only found in Mexico field ticks, for which larval packet DD data were not available. One collection (Rm28) appears to be an extreme outlier because it showed 0% survivorship in the DD assays (at both AI concentrations), yet the resistant genotype frequencies were similar to nearby collections from Zapata county that exhibit permethrin resistance (Rm26, Rm27, Rm29, Rm30, and Rm32) (Additional file 1: Table S1).

## Discussion

Our survey of *R. microplus* from the U.S. and Mexico field collections revealed three resistance SNPs in domains II and III of the *para*-sodium channel gene. To our knowledge, this is the first report of *para*-sodium channel resistance in *R. microplus* from the U.S. and Mexico that is based upon mutations within domain II [25,26,29]. Furthermore, we discovered a putative *super-kdr* SNP in domain II (T170C) [29] that has never been described previously in any tick species. Interestingly, we did not detect an abundance of the domain III SNP (T2134A) in the U.S. field collections as expected from previous studies [17,32,41]. However, this SNP was common in the Mexico field collections. This bias in U.S. collections may be due simply to stochastic effects caused by the movement of resistant ticks on wildlife, stray and illegal livestock, and imported cattle. Additionally, many of the U.S. resistant collections (13 of 21) are members of a distinct genetic group that is thought to have originated outside of Texas, most likely in Mexico [14]. This may explain the unexpected presence and high frequency of domain II SNPs (C190A and T170C) in the U.S. Although previous studies have suggested that the distribution of pyrethroid resistance SNPs in *R. microplus* is based primarily on geography [26,32], we found all three resistance SNPs in the relatively small area of the TEQA in southern Texas.

Previous descriptions of the *super-kdr* mutation M918T (T170C) in other arthropod species have always reported it in combination with other domain II mutations [29-31], however, we detected this SNP in all possible genotype pairings (Table 2). This suggests that T170C is independently associated with pyrethroid resistance in *R. microplus*. We observed this SNP in a homozygous state (MLG 4) in a handful of individuals (*n* = 5) from four collections (Figure 2). In addition, a single resistant tick was identified in field collection Rm34 that contained only the T170C mutation in heterozygous form and the corresponding F_1_ generation 0.125% AI larval packet DD data showed evidence of permethrin resistance, albeit at a low level (Additional file 1: Table S1). Furthermore, we detected the T170C SNP in a heterozygous state with the domain III T2134A SNP (MLG 6) in two Mexico field collections (Rm75 and Rm76). In cases where the T170C SNP was found in combination with C190A (MLG 2), cloning and sequencing revealed that these domain II mutations are not linked (Additional file 5: Figure S2 and Additional file 6: Figure S3). These are important findings because this mutation has never been reported to occur independently or in combination with a domain III mutation in any other arthropod species. However, since this is the first description of the T170C SNP [29] in *R. microplus* ticks, validation in experimental laboratory strains will be required to show if this is truly a *super-kdr* mutation. Interestingly, it has been experimentally shown *in vitro* that this mutation alone provides sufficient resistance to abolish pyrethroid sensitivity in the house fly [42]. It is possible this is also the case in *R. microplus*, and may suggest that ticks respond in a similar way to the selection pressure imposed by pyrethroids.

We observed a strong correlation between the proportion of individuals within a collection that carried any two resistance SNPs and the percent survivorship in permethrin larval packet DD assays (Figure 2). The regression analysis is consistent with a linear relationship at permethrin levels of 0.250% (Figure 3) and was corroborated by two independent datasets, one of which included laboratory ticks subjected to permethrin selection (Corrales F_4_). The population-level association of F_1_ phenotypes with a subsample of parental genotypes from field-collected adults clearly suggests that survival against permethrin should be stronger for individuals that carry two resistance SNPs. The coefficient of determination is probably a conservative lower-limit and we predict that if phenotype and genotype could be obtained from the same individual ticks, an even stronger association would exist. As an example, phenotype-genotype data were collected from the same generation of larvae (although not from the same individuals) in the Morgan *et al*. study, and these Australian populations demonstrate a substantial increase in the coefficient of determination (R^2^ = 0.9847) [25]. The variability we observed in a few field collections (such as Rm28) may have resulted from a lack of concordance between the genotypes of ticks used for SNP typing versus those used to create the F_1_ generation. We cannot exclude the possibility that the field-collected females used to produce F_1_ larvae for Rm28 were a biased sample of susceptible ticks that led to the deviation from expected resistance levels.

Our analyses suggest that the combination of any two resistance SNPs confers a similar and predictable level of resistance in *R. microplus* and, furthermore, that the application of insufficient permethrin concentrations may lead to increased resistance levels in tick populations. We observed a strong association regardless of the MLG present in a collection, which suggests that these SNPs may act additively and independently of each other but result in similar phenotypes. We acknowledge that the regression we observed might be predictive only at concentrations of permethrin close to 0.250% AI. Indeed, the association was lower at 0.125% AI (data not shown), possibly because the lower concentration of permethrin allowed many single-SNP heterozygotes to survive. If so, using a lower concentration of permethrin to treat cattle could increase the frequency of resistance SNPs in tick populations. Over time, ticks with double-SNP genotypes will inevitably appear and result in tick infestations with greater resistance levels to permethrin.

Since we never detected more than two resistance SNPs in any single *R. microplus* tick in this study (*n* = 1488), it is possible that a fitness cost may be associated with these SNPs that prevents individuals from accumulating more than two resistance mutations. Previous studies have reported a fitness cost associated with target site insensitivity to pyrethroids in house flies (*Musca domestica*) [43] and horn flies (*Haemotobia irritans*) [44,45]. Furthermore, it has been shown that in the absence of pyrethroid insecticide pressure, populations of both fly species revert to a less resistant state [30,43]. Interestingly, we detected an abundance of ticks from U.S. field collections that carried resistance SNPs (in heterozygous form) from multiple loci. These observations may suggest that there is an inherent fitness cost associated with the accumulation of multiple resistance SNPs within a single allele that could lead to homozygosity at more than one SNP locus. A heterozygous fitness advantage appears to exist for pyrethroid resistance mechanisms in voltage-gated sodium channel genes in other arthropod species [46,47]. Although pyrethroid selection pressure is probably low in southern Texas, we observed a high proportion of individuals with two heterozygous SNPs (MLG 2) in certain field collections, which suggests the accumulation of two resistance SNPs at different loci might not incur a fitness cost in *R. microplus*. If this is true, it may explain how these resistance mutations are able to persist in field populations that are not subject to pyrethroid selection pressure.

The exact source of pyrethroid resistance SNPs in Texas remains unknown, but we find it unlikely that these SNPs arose in Texas. First, pyrethroid acaricides are not used by the CFTEP for tick management in the TEQA and TPQAs. Second, field collections of Texas ticks were in Hardy-Weinberg equilibrium, which suggests the source populations had not experienced selection pressure from pyrethroids. An alternative explanation is that these SNPs have been introduced from Mexico (or another country) where pyrethroid acaricides are widely used. It is possible that ticks resistant to multiple acaricide classes [21,22] survived coumaphos treatments at the USDA ports of entry and were transported beyond the TEQA. This scenario is more likely for tick infestations that were discovered farther north of the TEQA, such as Rm20-Rm32. A recent study has shown that these 12 collections were sampled from a distinct genetic group of ticks in southern Texas [14]. Furthermore, low-levels of coumaphos resistance were reported for Rm21, Rm27, and Rm32 in this same study. Both lines of evidence are consistent with the long-distance transport of multi-acaricide resistant ticks from outside of southern Texas. In contrast, permethrin-resistant tick populations closer to the Rio Grande may have crossed the river on alternative hosts such as white-tailed deer. Repeated introductions via these two main routes have probably taken place, as suggested by the presence of three independent resistance mutations in different parts of the TEQA. These introductions have the potential to spread resistance SNPs widely in southern Texas via white-tailed deer and the movement of unregulated/illegal cattle.

During our query of the exons encoding domains II and III of the *para*-sodium channel gene, we came across five additional SNPs that do not appear to be associated with resistance (see Table 1). Some of these SNPs were found only in resistant collections, whereas others only occurred in susceptible populations. Therefore, we chose not to further investigate them but we present them here for the benefit of other interested researchers. The most intriguing SNP that may warrant further inquiry is the rare C190G SNP that occurs at the same nucleotide position as the known resistance C190A SNP. This mutation results in a leucine to valine substitution instead of the leucine/isoleucine change caused by the C190A SNP. The C190G mutation was found in a single collection from Mexico (Rm77) that also carries the C190A and T2134A mutations.

## Conclusions

Understanding the evolution of acaricide resistance that results from human-induced selection is clearly important for tick programs that depend on chemical control. In this study we have shown that novel resistance SNPs are present in the U.S. and Mexico, which sheds new light on the evolution of ticks in North America and other parts of the world where cattle fever ticks have invaded. Currently, characterization of resistant tick populations relies on bioassays. This biological test is an important first step because it provides phenotype data on the level of resistance in F_1_ larvae of field ticks. However, it is also time consuming and requires an adequate sample of live ticks and a facility licensed to rear F_1_ larvae; there are very few laboratories able to perform this kind of study. That said, many laboratories now have the capacity to perform DNA-based analyses. Our new PCR assays allowed us to rapidly screen almost 2,000 samples from two tick species, many of which had not been characterized by bioassay. Through this effort we were able to identify resistance SNPs in eight field collections that were not suspected to be resistant because larval packet DD data were not available. In one case, we found resistance SNPs on single ticks sampled from two white-tailed deer. This demonstrates the importance of running PCR assays for collections missing larval packet DD data due to small sample size, complications during rearing F_1_ larvae, or funding limitations.

We have demonstrated that molecular tools in combination with a simple linear regression model provide a powerful way to rapidly predict permethrin resistance levels in *R. microplus* ticks. This approach could be widely useful for maintaining the effectiveness of permethrin by avoiding its use on tick populations that already have a high frequency of resistance SNPs and are at risk of allele fixation. As in other parts of the world, the CFTEP relies almost exclusively on acaricide treatment to prevent the movement of cattle fever ticks outside of the TEQA. As such, acaricide resistance poses a serious threat to the success of the tick eradication program in southern Texas. The ability to rapidly detect resistance SNPs and ascertain the mechanisms of resistance in field populations of cattle fever ticks provides a powerful tool to help direct the effort of tick eradication and/or control programs.

## Additional files

Additional file 1: Table S1. Sampling information, genotype frequencies, and larval packet discriminating dose (DD) survival for cattle fever ticks sampled in Texas and Mexico. Additional file 2: Table S2. Real-time qPCR assays were developed for *Rhipicephalus microplus* based on the Melt-MAMA platform [38] to rapidly screen thousands of ticks for resistance SNPs at three loci in the *para*-sodium channel gene. Additional file 3: Figure S1. Methods for rapid detection Melt-MAMA [38] assays for three resistance SNP loci. Additional file 4: Figure S4. Agarose-MAMA [38] assays for *Rhipicephalus microplus* enable screening three resistance SNP loci in the *para*-sodium channel gene without the need for a real-time qPCR instrument. Additional file 5: Figure S2. Nucleotide sequence alignment for the exon encoding domain II from *Rhipicephalus microplus* mRNA sequence (putative sodium channel accession number: [GenBank:AF134216.2]) with two resistance alleles and three additional fly species that are known to harbour *kdr* and *super-kdr* mutations. Additional file 6: Figure S3. Amino acid sequence alignment for domain II from *Rhipicephalus microplus* (putative sodium channel accession number: AAD23600.2) with two resistance alleles and three additional fly species that are known to harbour L925I (*kdr*) and M918T (*super-kdr*) substitutions.
