# Supplementary material for: Multiple mutations in the para-sodium channel gene are associated with pyrethroid resistance in Rhipicephalus microplus from the United States and Mexico
Source: Parasit Vectors. 2014 Oct 1;7:456. doi: 10.1186/s13071-014-0456-z (PMC4189684; doi:10.1186/s13071-014-0456-z)
Supplement: Additional file 1: Table S1. — Sampling information, genotype frequencies, and larval packet discriminating dose (DD) survival for cattle fever ticks sampled in Texas and Mexico. [file 13071_2014_456_MOESM1_ESM.pdf]

Additional file 1: Table S1. Sampling information, genotype frequencies, and larval packet discriminating dose (DD) survival for cattle fever ticks sampled in Texas and Mexico. A total of 1488 *Rhipicephalus microplus* from 77 field collections were sampled from 60 properties; a single collection is defined as a sample of ticks from one property taken within a 30-day time window. Tick samples collected from the same property but separated by >30 days are treated as distinct collections. The host column includes domestic cattle from Texas or Mexico (cow), feral Mexican cattle that were captured on the Texas side of the U.S.-Mexico border (cow^{MX}), and white-tailed deer (WTD). Bold font is used to highlight the frequency of resistance SNPs and the percentage of survival in larval packet DD assays (Rm01-Rm63 DD data from Busch et al. [1]). We included permethrin-resistant laboratory tick strains as control populations. A total of 434 *Rhipicephalus annulatus* from 26 collections were sampled from 18 properties as described above. Hosts for *R. annulatus* include the same as *R. microplus* (cattle and WTD) plus exotic red deer (ERD), domestic U.S. horses (horse), and stray Mexican horses captured on the Texas side of the U.S-Mexico border (horse^{MX}). The three resistance loci within the *para*-sodium channel gene are named according to nucleotide position, following the convention of previous studies (see details in footnotes).

						C190A[a]			T170C[b]			T2134A[c]				% Survival in DD LPT[d]	
**Collections**	**Property**	**Collection Date**	**n**	**Host**	**County/ State**	**SS**	**RS**	**RR**	**SS**	**RS**	**RR**	**SS**	**RS**	**RR**	**Freq w/2 R SNPs**	**0.125%[e]**	**0.250%[e]**
**U.S. Field *R. microplus***																	
Rm01	RmProp01	3/10/2008	6	cow^{MX}	Webb	1	0	0	1	0	0	1	0	0	0	0	0
Rm02	RmProp02	10/9/2009	17	cow	Webb	1	0	0	1	0	0	1	0	0	0	0	0
Rm03	RmProp03	1/2/2008	6	cow	Webb	1	0	0	1	0	0	1	0	0	0	0	0
Rm04	RmProp04	12/2/2009	6	cow	Webb	1	0	0	1	0	0	1	0	0	0	0	0
Rm05	RmProp05	6/19/2008	30	cow	Zapata	1	0	0	1	0	0	1	0	0	0	0	0
Rm06	RmProp06	12/17/2009, 01/07/2010	29	WTD	Zapata	1	0	0	1	0	0	1	0	0	0	0	0
Rm07	RmProp07	1/21/2010	7	WTD	Zapata	1	0	0	1	0	0	1	0	0	0	0	0
Rm08	RmProp08	12/9/2009	10	WTD	Zapata	1	0	0	1	0	0	1	0	0	0	0	0
Rm09	RmProp09	11/14/2007	14	cow	Zapata	1	0	0	1	0	0	1	0	0	0	0	0
Rm10	RmProp10	5/6/2009, 06/04/2009	31	cow	Zapata	1	0	0	1	0	0	1	0	0	0	0	0
Rm11	RmProp10	9/28/2009	11	cow	Zapata	1	0	0	1	0	0	1	0	0	0	0	0
Rm12	RmProp11	6/20/2007	27	cow	Zapata	1	0	0	1	0	0	1	0	0	0	0	0
Rm13	RmProp11	7/27/2009	20	cow	Zapata	1	0	0	1	0	0	1	0	0	0	0	0
Rm14	RmProp11	11/16/2009	13	WTD	Zapata	1	0	0	1	0	0	1	0	0	0	0	0
Rm15	RmProp12	12/4/2008	12	WTD	Zapata	1	0	0	1	0	0	1	0	0	0	0	0
Rm16	RmProp13	12/28/2009, 01/13/2010	20	WTD	Zapata	1	0	0	1	0	0	1	0	0	0	0	0
Rm17	RmProp14	7/16/2009	20	cow	Zapata	1	0	0	1	0	0	1	0	0	0	0	0
Rm18	RmProp15	5/19/2009	6	cow	Zapata	1	0	0	1	0	0	1	0	0	0	0	0
Rm19	RmProp16	5/6/2009	2	cow	Zapata	1	0	0	1	0	0	1	0	0	0	N/A	N/A

Rm20	RmProp17	5/7/2008	2	WTD	Zapata	0	0	**1**	1	0	0	1	0	0	1	N/A	N/A
Rm21	RmProp18	6/26/2008	4	cow	Zapata	0	0	**1**	1	0	0	1	0	0	1	**100**	**100**
Rm22	RmProp19	5/6/2009	2[HWE]	cow	Zapata	0.5	**0.5**	0	0	**1**	0	1	0	0	0.5	N/A	N/A
Rm23	RmProp20	5/6/2009	7[HWE]	cow	Zapata	0.571	**0.286**	**0.143**	0.714	**0.286**	0	1	0	0	0.143	N/A	N/A
Rm24	RmProp21	6/25/2009	1	cow	Zapata	0	0	**1**	1	0	0	1	0	0	1	N/A	N/A
Rm25	RmProp22	05/4/2009, 05/15/2009	6[HWE]	cow	Zapata	0	**0.333**	**0.667**	1	0	0	1	0	0	0.667	N/A	N/A
Rm26	RmProp23	11/5/2008	19[HWE]	cow	Zapata	0.105	**0.632**	**0.263**	0.632	**0.316**	**0.053**	1	0	0	0.579	**62.6**	**61.1**
Rm27	RmProp24	10/27/2008, 11/05/2008	24[HWE]	cow	Zapata	0.167	**0.292**	**0.542**	0.708	**0.25**	**0.042**	1	0	0	0.708	**73.6, 62.1**	**77.5, 64.7**
Rm28	RmProp25	4/15/2009, 4/24/2009, 05/06/2009	36[HWE]	cow	Zapata	0.194	**0.44**	**0.361**	0.667	**0.306**	**0.028**	1	0	0	0.556	0	0
Rm29	RmProp26	4/15/2009, 04/24/2009, 05/01/2009, 05/15/2009	25[HWE]	cow	Zapata	0.12	**0.52**	**0.36**	0.68	**0.32**	0	1	0	0	0.56	**45.2**	**16.6**
Rm30	RmProp27	4/20/2009, 04/24/2009, 05/01/2009	60[HWE]	cow	Zapata	0.217	**0.433**	**0.35**	0.617	**0.367**	**0.016**	1	0	0	0.633	**92.8**	**70.7**
Rm31	RmProp27	7/16/2009	12[HWE]	cow	Zapata	0.25	**0.25**	**0.5**	0.667	**0.25**	**0.083**	1	0	0	0.667	N/A	N/A
Rm32	RmProp28	6/26/2008	18[HWE]	cow	Zapata	0.111	**0.389**	**0.5**	0.611	**0.389**	0	1	0	0	0.833	**65.3**	**77.2**
Rm33	RmProp29	11/15/2007	7	cow[MX]	Starr	1	0	0	1	0	0	1	0	0	0	0	0
Rm34	RmProp30	4/29/2005, 05/03/2005	42[HWE]	cow	Starr	1	0	0	0.976	**0.024**	0	1	0	0	0	**0.5**	0
Rm35	RmProp31	6/6/2008	22	cow	Starr	1	0	0	1	0	0	1	0	0	0	N/A	N/A
Rm36	RmProp32	12/30/2008	31[HWE]	WTD	Starr	1	0	0	1	0	0	0.935	**0.065**	0	0	N/A	N/A
Rm37	RmProp33	3/27/2008	15	cow	Starr	1	0	0	1	0	0	1	0	0	0	N/A	N/A
Rm38	RmProp34	5/23/2008	24	cow	Starr	1	0	0	1	0	0	1	0	0	0	N/A	N/A
Rm39	RmProp34	3/18/2009	18	cow	Starr	1	0	0	1	0	0	1	0	0	0	N/A	N/A
Rm40	RmProp34	4/9/2009, 04/24/2009	14	cow	Starr	1	0	0	1	0	0	1	0	0	0	N/A	N/A
Rm41	RmProp35	6/4/2008	13	WTD	Starr	1	0	0	1	0	0	1	0	0	0	N/A	N/A
Rm42	RmProp36	2/22/2008	30	cow	Starr	1	0	0	1	0	0	1	0	0	0	0	0
Rm43	RmProp37	4/11/2008	48	cow	Starr	1	0	0	1	0	0	1	0	0	0	N/A	N/A
Rm44	RmProp38	5/19/2009	16	cow	Starr	1	0	0	1	0	0	1	0	0	0	0	0
Rm45	RmProp38	7/27/2009	7	cow	Starr	1	0	0	1	0	0	1	0	0	0	0	0
Rm46	RmProp39	2/17/2009	30	cow	Starr	1	0	0	1	0	0	1	0	0	0	0	0
Rm47	RmProp39	3/3/2009	171	WTD	Starr	1	0	0	1	0	0	1	0	0	0	N/A	N/A
Rm48	RmProp39	3/4/2009	22	cow	Starr	1	0	0	1	0	0	1	0	0	0	N/A	N/A
Rm49	RmProp40	4/2/2009	29	cow	Starr	1	0	0	1	0	0	1	0	0	0	0	0
Rm50	RmProp41	6/19/2008	56[HWE*]	cow	Starr	0.982	0	**0.018**	1	0	0	1	0	0	0.018	**0.8**	**1.5**
Rm51	RmProp42	1/16/2009	30	cow	Starr	1	0	0	1	0	0	1	0	0	0	0	0
Rm52	RmProp42	2/27/2009	30	cow	Starr	1	0	0	1	0	0	1	0	0	0	0	0
Rm53	RmProp42	3/27/2009	11	cow	Starr	1	0	0	1	0	0	1	0	0	0	N/A	N/A
Rm54	RmProp42	4/10/2009	10	cow	Starr	1	0	0	1	0	0	1	0	0	0	N/A	N/A
Rm55	RmProp42	5/4/2009	3	cow	Starr	1	0	0	1	0	0	1	0	0	0	N/A	N/A
Rm56	RmProp42	7/8/2009	20	cow	Starr	1	0	0	1	0	0	1	0	0	0	N/A	N/A
Rm57	RmProp42	8/13/2009	2	cow	Starr	1	0	0	1	0	0	1	0	0	0	N/A	N/A
Rm58	RmProp43	5/4/2009	18	cow	Starr	1	0	0	1	0	0	1	0	0	0	N/A	N/A
Rm59	RmProp44	4/15/2009	30	cow	Starr	1	0	0	1	0	0	1	0	0	0	0	0
Rm60	RmProp45	5/6/2009	15	cow	Starr	1	0	0	1	0	0	1	0	0	0	N/A	N/A
Rm61	RmProp45	6/8/2009, 06/29/2009	3	cow	Starr	1	0	0	1	0	0	1	0	0	0	N/A	N/A

Rm62	RmProp45	7/31/2009	1	cow	Starr	1	0	0	1	0	0	1	0	0	0	N/A	N/A
Rm63	RmProp46	5/5/2008	3	cow	Hidalgo	1	0	0	1	0	0	1	0	0	0	N/A	N/A
Rm64[S]	RmProp47	9/17/2010, 9/24/2010, 10/1/2010	4[HWE*]	cow	Hidalgo	0	**1**	0	1	0	0	1	0	0	0	**34.1**	**16.5**
Rm65[S]	RmProp48	11/8/2011	29[HWE]	cow[MX]	Cameron	0.621	**0.345**	**0.034**	1	0	0	0.821	**0.179**	0	0.103	**44**	**19.1**
Rm66[S]	RmProp49	6/11/2010, 7/23/2010, 7/30/2010	26[HWE]	cow	Starr	0.231	**0.423**	**0.346**	1	0	0	1	0	0	0.346	**14.4**	**4.8**
Rm67[S]	RmProp50	1/5/2011	23[HWE]	cow	Starr	0.087	**0.435**	**0.478**	1	0	0	0.826	**0.174**	0	0.652	**86.1**	**76.3**
Rm68[S] (NVSL_T620)	RmProp51	6/16/2010	1	cow	Webb	0	**1**	0	1	0	0	1	0	0	0	N/A	N/A
**Mexico Field**																	
Rm69[S]	RmProp52	2012	1	cow	Colima	1	0	0	1	0	0	1	0	0	0	N/A	N/A
Rm70[S]	RmProp53	2012	1	cow	Chiapas	1	0	0	1	0	0	0	**1**	0	0	N/A	N/A
Rm71[S]	RmProp54	2012	2	cow	Chiapas	1	0	0	1	0	0	0	0	**1**	1	N/A	N/A
Rm72[S]	RmProp55	2012	2	cow	Colima	1	0	0	1	0	0	1	0	0	0	N/A	N/A
Rm73[S]	RmProp56	2012	2	cow	Colima	1	0	0	1	0	0	1	0	0	0	N/A	N/A
Rm74[S]	RmProp57	2012	2[HWE]	cow	Colima	0.5	**0.5**	0	1	0	0	0.5	**0.5**	0	0	N/A	N/A
Rm75[S]	RmProp58	2012	7[HWE]	cow	Hidalgo	0.571	**0.286**	**0.143**	0.714	**0.286**	0	0.143	**0.571**	**0.286**	1	N/A	N/A
Rm76[S]	RmProp59	2012	7[HWE]	cow	Veracruz	0.571	**0.429**	0	0.857	**0.143**	0	0.143	**0.571**	**0.286**	0.714	N/A	N/A
Rm77[S]	RmProp60	2012	7[HWE*]	cow	San Luis Potosi	0.429	0	**0.143**	1	0	0	0.857	**0.143**	0	0.143	N/A	N/A
**Laboratory Strains**																	
B&H Ranch	B&H Ranch	8/3/2004 (F6)	10	cow	Hidalgo	1	0	0	1	0	0	0	0	**1**	1	N/A	N/A
Corrales	Corrales	7/8/1996 (F4)	30	cow	Colima	1	0	0	1	0	0	0	0	**1**	1	**100**	**100**
San Felipe	San Felipe	10/26/2004 (F44)	30[HWE]	cow	Tamaulipas	0.867	**0.133**	0	1	0	0	0.067	**0.367**	**0.567**	0.7	N/A	N/A
Santa Luiza	Santa Luiza	3/22/2005 (F20)	30	cow	Brazil	0	0	**1**	1	0	0	1	0	0	1	N/A	N/A
**U.S. Field *R. annulatus***																	
Ra01	RaProp01	6/12/2008	1	cow	Val Verde	1	0	0	1	0	0	1	0	0	0	N/A	N/A
Ra02	RaProp02	3/3/2006	23	cow[MX]	Kinney	1	0	0	1	0	0	1	0	0	0	0	0
Ra03	RaProp03	3/9/2005	16	cow	Kinney	1	0	0	1	0	0	1	0	0	0	**4.5**	0
Ra04	RaProp03	12/4/2007	28	cow	Kinney	1	0	0	1	0	0	1	0	0	0	0	0
Ra05	RaProp04	12/28/2007	3	ERD	Maverick	1	0	0	1	0	0	1	0	0	0	N/A	N/A
Ra06	RaProp04	7/15/2008	24	ERD	Maverick	1	0	0	1	0	0	1	0	0	0	N/A	N/A
Ra07	RaProp04	6/16/2009	6	ERD	Maverick	1	0	0	1	0	0	1	0	0	0	0	0
Ra08	RaProp04	10/30/2009	31	ERD	Maverick	1	0	0	1	0	0	1	0	0	0	0	0
Ra09	RaProp04	5/24/2010	36	ERD	Maverick	1	0	0	1	0	0	1	0	0	0	0	0
Ra10	RaProp05	3/23/2007	17	cow[MX]	Maverick	1	0	0	1	0	0	1	0	0	0	0	0
Ra11	RaProp06	12/7/2006	11	cow	Maverick	1	0	0	1	0	0	1	0	0	0	0	0
Ra12	RaProp07	6/15/2007	10	cow	Maverick	1	0	0	1	0	0	1	0	0	0	0	0
Ra13	RaProp08	2/26/2010	8	horse	Maverick	1	0	0	1	0	0	1	0	0	0	N/A	N/A
Ra14	RaProp09	9/26/2008	19	cow	Maverick	1	0	0	1	0	0	1	0	0	0	N/A	N/A
Ra15	RaProp10	3/14/2008	21	cow	Maverick	1	0	0	1	0	0	1	0	0	0	0	0

Ra16	RaProp11	11/13/2007	8	cow	Dimmit	1	0	0	1	0	0	1	0	0	0	0	0
Ra17	RaProp11	6/2/2008, 6/27/2008	41	cow	Dimmit	1	0	0	1	0	0	1	0	0	0	0	0
Ra18	RaProp12	11/6/2007	30	cow	Dimmit	1	0	0	1	0	0	1	0	0	0	0	0
Ra19	RaProp13	8/7/2007	2	cow	Dimmit	1	0	0	1	0	0	1	0	0	0	0	0
Ra20	RaProp14	11/16/2007	30	cow	Webb	1	0	0	1	0	0	1	0	0	0	0	0
Ra21	RaProp14	11/30/2007	13	WTD	Webb	1	0	0	1	0	0	1	0	0	0	N/A	N/A
Ra22	RaProp15	3/7/2008	29	cow	Webb	1	0	0	1	0	0	1	0	0	0	N/A	N/A
Ra23	RaProp16	5/6/2008	21	cow	Webb	1	0	0	1	0	0	1	0	0	0	0	0
Ra24	RaProp17	6/4/2009	2	cow	Webb	1	0	0	1	0	0	1	0	0	0	N/A	N/A
Ra25	RaProp18	11/30/2007	3	cow	Webb	1	0	0	1	0	0	1	0	0	0	N/A	N/A
Ra26	RaProp19	5/4/2005	1	horse[MX]	Maverick	1	0	0	1	0	0	1	0	0	0	N/A	N/A

[a]C190A, Morgan et al. 2009; [b]T170C, Williamson et al. 1996; [c]T2134A, He et al. 1999; [d]FAO 1971.

[e]The USDA tests two levels of permethrin active ingredient (AI): 0.125% and 0.250%

[S]Collections added to the STRUCTURE population assignment analysis of 63 *R. microplus* collections described previously [1].

[HWE]Collections found to be in Hardy-Weinberg equilibrium (collections that were monomorphic and/or had a sample size of one were not tested).

[HWE*]Collections out of Hardy-Weinberg equilibrium.

1. Busch JD, Stone NE, Nottingham R, Araya-Anchetta A, Lewis J, Hochhalter C, Giles JR, Gruendike J, Freeman J, Buckmeier G *et al*: **Widespread movement of invasive cattle fever ticks (*Rhipicephalus microplus*) in southern Texas leads to shared local infestations on cattle and deer**. *Parasit Vectors* 2014, **7**(188):188.
