# Supplementary material for: Multiple mutations in the para-sodium channel gene are associated with pyrethroid resistance in Rhipicephalus microplus from the United States and Mexico
Source: Parasit Vectors. 2014 Oct 1;7:456. doi: 10.1186/s13071-014-0456-z (PMC4189684; doi:10.1186/s13071-014-0456-z)
Supplement: Additional file 2: Table S2. — Real-time qPCR assays were developed for Rhipicephalus microplus based on the Melt-MAMA platform [38] to rapidly screen thousands of ticks for resistance SNPs at three loci in the para-sodium channel gene. [file 13071_2014_456_MOESM2_ESM.pdf]

Additional file 2: Table S2. Real-time qPCR assays were developed for *Rhipicephalus microplus* based on the Melt-MAMA platform [1] to rapidly screen thousands of ticks for resistance SNPs at three loci in the *para*-sodium channel gene. PCRs were carried out in competitive reactions using SNP-specific forward primers designed to amplify all susceptible and resistance SNPs. Concentrations of the forward primers are skewed to equalize the amplification efficiency of both amplicons. Genotyping was achieved by generating a post-PCR dissociation curve and analyzing the melting temperature of target amplicons. All three qPCR assays accurately detected homozygous and heterozygous genotypes. Betaine was used as a controlled PCR inhibitor to increase target site specificity and minimize non-specific amplification. To balance the effects of the betaine, an extra 0.2 U of Platinum® *Taq* polymerase was added to each PCR to maintain the efficiency of these assays. All PCRs were carried out under the same conditions, with the exception of primer concentrations and annealing temperature (T_m). All three assays are also able to detect resistance SNPs in a related species, *Rhipicephalus annulatus*. Assay conditions for *R. annulatus* are identical to *R. microplus* with the exception of annealing temperature (T_m) for domain II (C190A), which is decreased to 58°C.

Assay	Locus	SNP (S/R)	Primer sequence (5'-3')	Primer concentrations	T_m	Dissociation Curve Temp.
**Domain II**	C190A	C/A	**Susceptible (RmNaDomainIIF1_ANC):** F-GGAAAACCATCGGTGCTC	0.09µM	60°C (58°C for Ra)	Susceptible: 72°C
			**Resistant (RmNaDomainIIF2_DER):** F-gcccgcccgcccgcccGGAAAACCATCGGTGACA	0.06µM		Resistant: 78°C
			**Conserved reverse (RmNaDomainIIR2_CON):** R-CCCAGGACAAAGGTGAAGT	0.15µM		
**Domain II (*super-kdr*)**	T170C	T/C	**Susceptible (RmNaSuperKDRF1_ANC):** F-CCTTAACCTGCTCATCTCTATCCT	0.10µM	63°C	Susceptible: 70°C
			**Resistant (RmNaSuperKDRF1_DER):** F-gcccgcccgcccgcccCCCTTAACCTGCTCATCTCTATCTC	0.05µM		Resistant: 78°C
			**Conserved reverse (RmNaSuperKDRR1_CON):** R-GCACCGATGGTTTTCCCC	0.15µM		
**Domain III**	T2134A	T/A	**Susceptible (RmNaDomainIIIF3_ANC):** F-TCATTATCTTCGGCTCCTCCT	0.09µM	57°C	Susceptible: 70°C
			**Resistant (RmNaDomainIIIF4_DER):** F-gcccgcccgcccgcccTCATTATCTTCGGCTCCTTGA	0.06µM		Resistant: 76°C
			**Conserved reverse (RmNaDomainIIIR2_CON):** R-CGATAATAACACCGATGAATAGA	0.15µM		
**Universal reagents (10µL reactions)**			20-40ng of DNA template<br>1X SYBR® Green Master Mix<br>1.2µM betaine<br>0.2U additional Platinum® *Taq* polymerase			
**Universal thermocycling conditions**			95°C for 10 minutes<br>**40 cycles of:**<br>Step 1: 95°C for 15 seconds,<br>Step 2: **T_m** for 60 seconds<br>Step 3: 72°C for 30 seconds			

1. Birdsell DN, Pearson T, Price EP, Hornstra HM, Nera RD, Stone N, Gruendike J, Kaufman EL, Pettus AH, Hurbon AN *et al*: **Melt analysis of mismatch amplification mutation assays (Melt-MAMA): a functional study of a cost-effective SNP genotyping assay in bacterial models**. *PloS one* 2012, **7**(3):e32866.
