# Supplementary material for: Multiple mutations in the para-sodium channel gene are associated with pyrethroid resistance in Rhipicephalus microplus from the United States and Mexico
Source: Parasit Vectors. 2014 Oct 1;7:456. doi: 10.1186/s13071-014-0456-z (PMC4189684; doi:10.1186/s13071-014-0456-z)
Supplement: Additional file 3: Figure S1. — Methods for rapid detection Melt-MAMA [38] assays for three resistance SNP loci. [file 13071_2014_456_MOESM3_ESM.pdf]

Additional file 3: Figure S1. Methods for rapid detection Melt-MAMA [1] assays for three resistance SNP loci. The first SNP we targeted (C190A) is located in the exon encoding domain II of the *para*-sodium channel gene and corresponds to nucleotide position 190 in *R. microplus* mRNA sequence (putative sodium channel accession number [GenBank:AF134216.2]). We developed specific forward primers to detect a cytosine for the susceptible (ancestral) SNP RmNaDomainIIF1_ANC (5'GGAAAACCATCGGTGCTC) or an adenine for the resistance (derived) SNP RmNaDomainIIF2_DER (5'gcccgcccgcccgcccGGAAAACCATCGGTGACA). The repeated GC motif in the tail of the derived forward primer increases the amplicon melting temperature compared to the ancestral primer, thus providing an efficient way to distinguish the susceptible vs. resistance SNPs. The reverse primer RmNaDomainIIR2_CON (5'CCCAGGACAAAGGTGAAGT) is conserved for both SNPs, thus the amplicon lengths are 59 bp for the resistant product and 43 bp for the susceptible product. All three primers were used in a competitive PCR; reactions were carried out in 10 µL volumes containing the following reagents (given in final concentrations): 20-40 ng of DNA template, 1x SYBR® Green Master Mix (Invitrogen, Carlsbad, CA, USA), 1.2 µM betaine to increase target specificity, 0.2 U Platinum *Taq* DNA Polymerase, 0.09 µM of forward primer RmNaDomainIIF1_ANC, 0.06 µM of forward primer RmNaDomainIIF2_DER, and 0.15 µM of reverse primer RmNaDomainIIR2_CON. Quantitative PCRs were run on an AB7500 instrument (Applied Biosystems, Foster City, CA, USA) under the following thermocycling conditions: 95°C for 10 minutes to release the polymerase antibody, followed by 40 cycles of 95°C for 15 seconds, 60°C for 60 seconds, and 72°C for 30 seconds. Subsequently, a dissociation curve was generated for each sample. Due to the GC tail on primer RmNaDomainIIF2_DER, the resistant product

dissociated at 78°C, while the susceptible product dissociated at 72°C (Fig. S1a).

a)

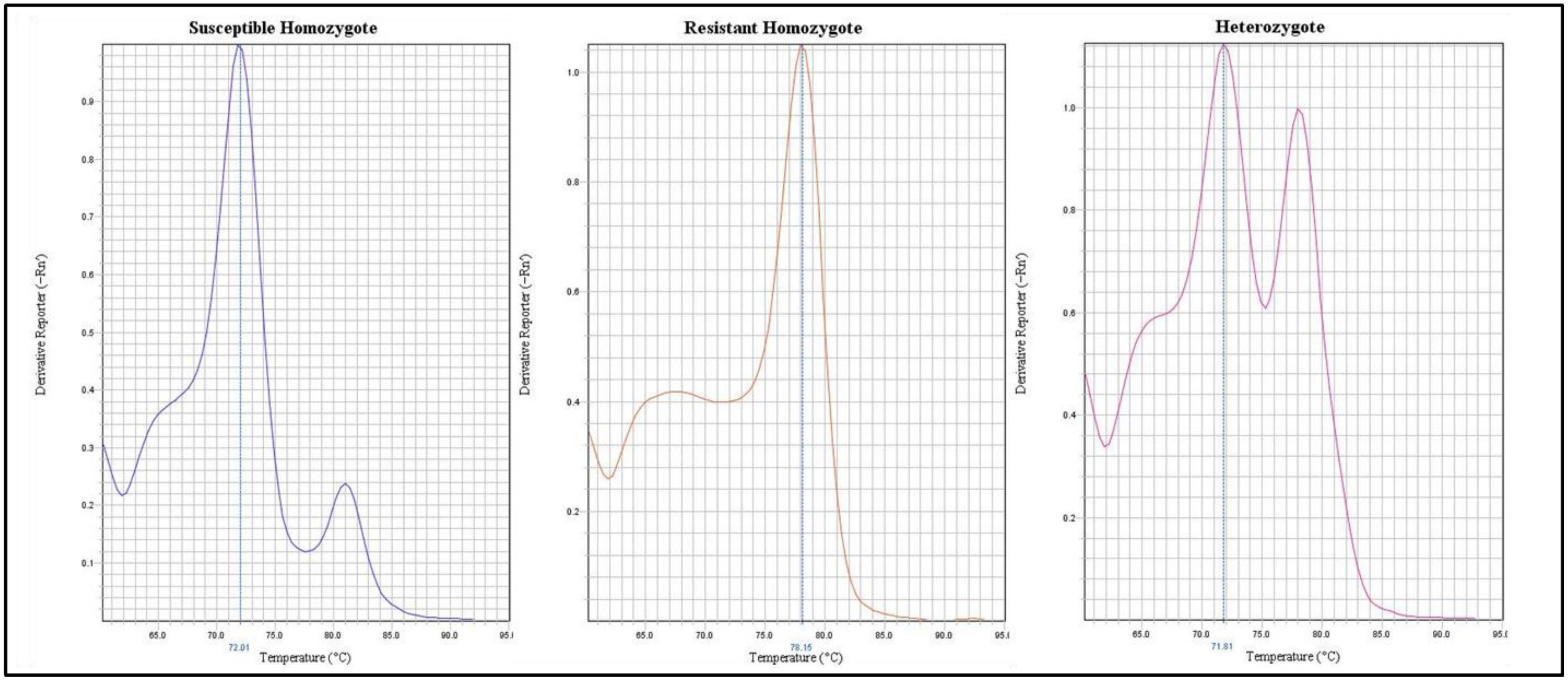

The second SNP we targeted (T170C) is also located in domain II and corresponds to position 170 of the *R. microplus para*-sodium channel gene mRNA sequence [GenBank:AF134216.2]. This SNP has been described in other species and is known to provide *super-kdr* resistance [29]. To our knowledge, it has not been described previously in any tick species. We developed specific forward primers to detect a thymine for the susceptible SNP RmNaSuperKDRF1_ANC (5'CCTTAACCTGCTCATCTCTATCCT) or a cytosine for the resistance SNP RmNaSuperKDRF1_DER (5'gcccgcccgcccgcccCCCTTAACCTGCTCATCTCTATCTC). The conserved reverse primer RmNaSuperKDRR1_CON (5'GCACCGATGGTTTTCCCC) resulted in amplicon lengths of 59 bp for the resistant product and 42 bp for the susceptible product. All three primers were used in the previously described competitive PCR, with

0.1 µM of forward primer RmNaSuperKDRF1_ANC, 0.05 µM of forward primer RmNaSuperKDRF1_DER, and 0.15 µM of reverse primer RmNaSuperKDRR1_CON. Quantitative PCRs were run under the previously described thermocycling conditions, with the exception of a change in annealing temperature to 63°C. Due to the GC tail on primer RmNaSuperKDRF1_DER, the resistant product dissociates at 78°C, while the susceptible product dissociates at 70°C (Fig. S1b).

b)

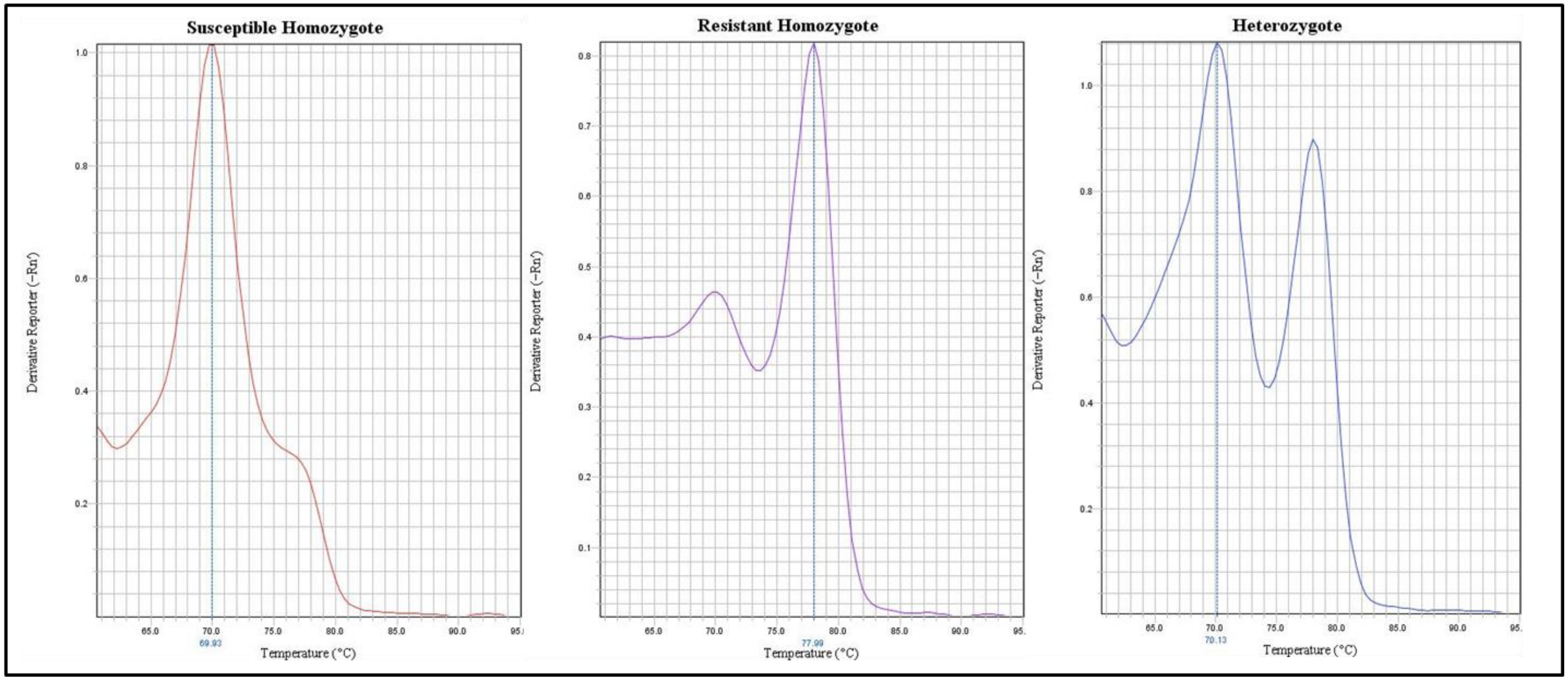

The third SNP we targeted (T2134A) is located in domain III of the *para*-sodium channel gene and corresponds to position 2134 in *R. microplus* mRNA sequence [GenBank:AF134216.2]. We developed specific forward primers to detect a thymine for the susceptible SNP RmNaDomainIIIF3_ANC (5'TCATTATCTTCGGCTCCTCCT) or an adenine for the resistance SNP RmNaDomainIIIF4_DER (5'gcccgcccgcccgcccTCATTATCTTCGGCTCCTTGA). The conserved reverse primer RmNaDomainIIIR2_CON (5'CGATAATAACACCGATGAATAGA) resulted in amplicon lengths of 70 bp for the resistant product and 54 bp for the susceptible product. All three primers were used in the previously described competitive PCR, with 0.09 µM of

forward primer RmNaDomainIIIF3_ANC, 0.06 μM of forward primer RmNaDomainIIIF4_DER, and 0.15 μM of reverse primer RmNaDomainIIIR2_CON. Quantitative PCRs were run under the previously described conditions, with the exception of a change in annealing temperature to 57°C. Due to the GC tail on primer RmNaDomainIIIF4_DER, the resistant product dissociated at 76°C, while the susceptible product dissociated at 70°C (Fig. S1c). We ran resistant and susceptible controls for all three assays (see Additional file 2: Table S2 for all qPCR assay details).

c)

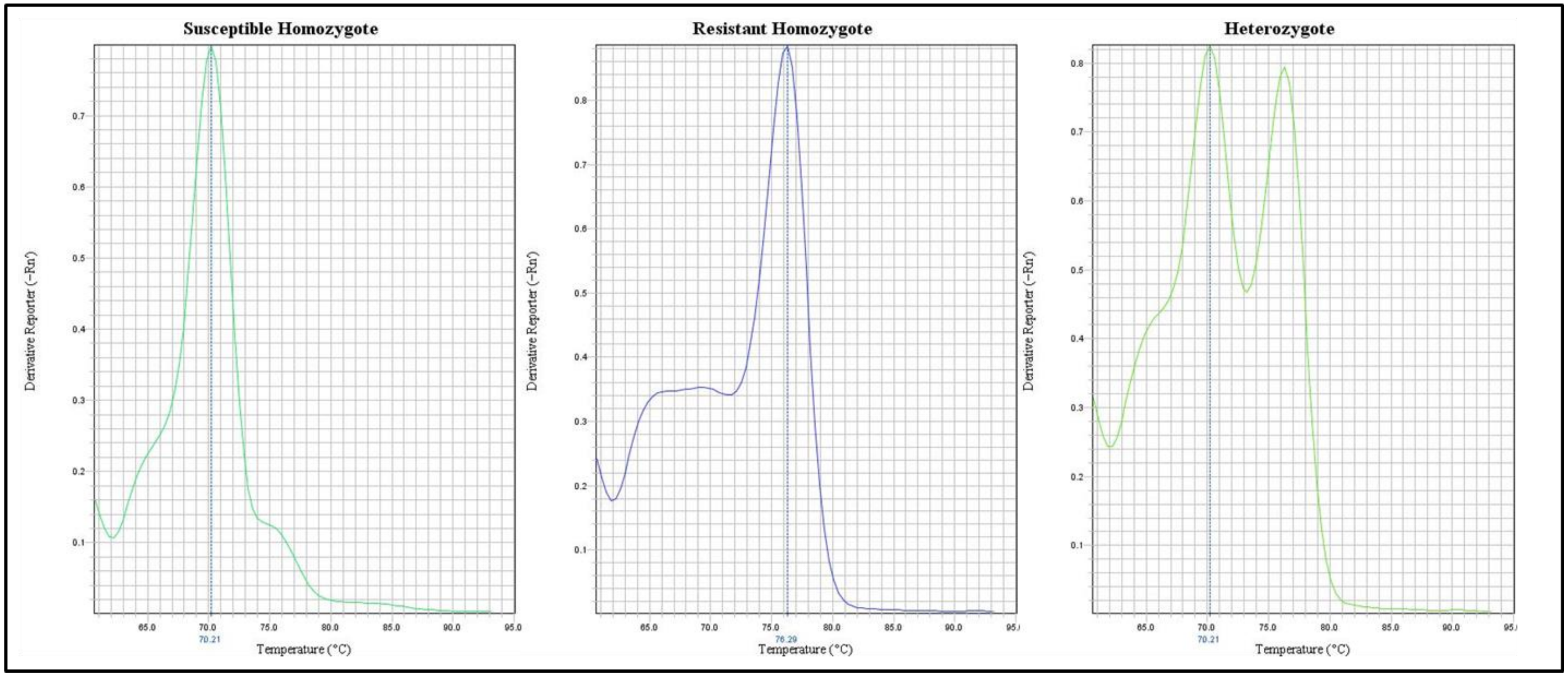

1. Birdsell DN, Pearson T, Price EP, Hornstra HM, Nera RD, Stone N, Gruendike J, Kaufman EL, Pettus AH, Hurbon AN *et al*: **Melt analysis of mismatch amplification mutation assays (Melt-MAMA): a functional study of a cost-effective SNP genotyping assay in bacterial models**. *PloS one* 2012, **7**(3):e32866.
