# Supplementary material for: Multiple mutations in the para-sodium channel gene are associated with pyrethroid resistance in Rhipicephalus microplus from the United States and Mexico
Source: Parasit Vectors. 2014 Oct 1;7:456. doi: 10.1186/s13071-014-0456-z (PMC4189684; doi:10.1186/s13071-014-0456-z)
Supplement: Additional file 4: Figure S4. — Agarose-MAMA [38] assays for Rhipicephalus microplus enable screening three resistance SNP loci in the para-sodium channel gene without the need for a real-time qPCR instrument. [file 13071_2014_456_MOESM4_ESM.pdf]

Additional file 6: Figure S4. Agarose-MAMA [1] assays for *Rhipicephalus microplus* enable screening three resistance SNP loci in the *para*-sodium channel gene without the need for a real-time qPCR instrument. The conversion of three qPCR Melt-MAMA assays to a conventional platform (standard PCR and agarose gels) required several modifications at each locus. The C190A domain II assay utilized forward primers RmNaDomainIIF1_ANC and RmNaDomainIIF2_DER and reverse primer RmNaDomainIIR2_CON, which are the same primers used in the Melt-MAMA qPCR (Additional file 2: Table S2). The repeated GC motif in the tail of the derived forward primer increases the amplicons molecular weight by 16 bp compared to the ancestral primer, thus providing an effective way to distinguish the susceptible vs. resistance SNPs based on amplicon length. As the reverse primer is conserved for both SNPs, the amplicon lengths are 59 bp for the resistant product and 43 bp for the susceptible product. All three primers were used in a competitive PCR; reactions were carried out in 10 µL volumes containing the following reagents (given in final concentrations): 20-40 ng of DNA template, 1x PCR buffer, 2.0 mM $MgCl_2$, 0.3 mM dNTPs, 0.8 U Platinum® *Taq* polymerase, 0.09 µM of forward primer RmNaDomainIIF1_ANC, 0.06 µM of forward primer RmNaDomainIIF2_DER, and 0.15 µM of reverse primer RmNaDomainIIR2_CON. PCRs were thermocycled according to following conditions: 95°C for 10 minutes to release the polymerase antibody, followed by 40 cycles of 94°C for 30 seconds, 55°C for 30 seconds, and 72°C for 30 seconds. Conventional PCRs were run on a DNA Engine (MJ Research, Watertown, MA, USA) and visualized on 2.5% agarose gel using 20x lithium borate buffer, which was determined to be an excellent agarose gel buffer for providing high resolution for low molecular weight amplicons. Gel electrophoresis was carried out using 10 µL of PCR product with 3 µL of 6x xylene cyanol loading dye and electrophoresed for 30min at 250V.

The T170C domain II assay utilized forward primers RmNaSuperKDRF1_ANC and RmNaSuperKDRF1_DER-long (gcccgcccgcccgcccgcccgcccCCCTTAACCTGCTCATCTCTATCTC) and reverse primer RmNaSuperKDRR1_CON, which are the same primers used in the Melt-MAMA qPCR assay (Additional file 2: Table S2), except the forward primer RmNaSuperKDRF1_DER-long incorporates an additional eight base pairs in the GC motif to maximize the separation of resistant and susceptible amplicons during gel electrophoresis. As the reverse primer is conserved for both SNPs, the amplicon lengths are 67 bp for the resistant product and 42 bp for the susceptible product. All three primers were used in a competitive PCR; reactions were carried out in 10 µL volumes containing the following reagents (given in final concentrations): 20-40 ng of DNA template, 1x PCR buffer, 2.5 mM $MgCl_2$, 0.2 mM dNTPs, 0.5 µg/µL bovine serum albumin (BSA) to increase PCR yield, 0.8 U Platinum® *Taq* polymerase, 0.125 µM of forward primer RmNaSuperKDRF1_ANC, 0.05 µM of forward primer RmNaSuperKDRF1_DER-long, and 0.15 µM of reverse primer RmNaSuperKDRR1_CON. PCRs were thermocycled according to following conditions: 95°C for 10 minutes to release the polymerase antibody, followed by 38 cycles of 94°C for 30 seconds, 68°C for 30 seconds, and 72°C for 30 seconds. Gel electrophoresis was carried out as described above.

The T2134A domain III assay utilized forward primers RmNaDomainIIIF3_ANC and RmNaDomainIIIF4_DER-long (gcccgcccgcccgcccgcccgcccTCATTATCTTCGGCTCCTTGA) and reverse primer RmNaDomainIIIR2_CON, which are the same primers used in the Melt-MAMA qPCR assay (Additional file 2: Table S2), except the forward primer RmNaDomainIIIF4_DER-long incorporates an additional eight base pairs in the GC motif to maximize separation of resistant and susceptible amplicons during gel electrophoresis. As the reverse primer is conserved for both SNPs, the amplicon lengths are 78 bp for the resistant product and 54 bp for the susceptible product. All three primers were used in a competitive PCR; reactions were carried out in 10 µL volumes containing the following reagents (given in final concentrations): 20-40 ng of DNA template, 1x PCR buffer, 3.0 mM $MgCl_2$, 0.3 mM dNTPs, 1.2 µM betaine to increase target specificity, 1 U Platinum® *Taq* polymerase, 0.15 µM of forward primer RmNaDomainIIIF3_ANC, 0.15 µM of forward primer RmNaDomainIIIF4_DER-long, and 0.15 µM of reverse primer RmNaDomainIIIR2_CON. PCRs were thermocycled according to following conditions: 95°C for 10 minutes to release the polymerase antibody, followed by 40 cycles of 94°C for 30 seconds, 61°C for 30 seconds, and 72°C for 30 seconds. Gel electrophoresis was carried out as described above. All three Agarose-MAMA SNP assays were validated using a subset of 20 ticks that were representative of all possible genotypes at each locus (data not shown). An example of each genotype is provided below (Fig. S4).

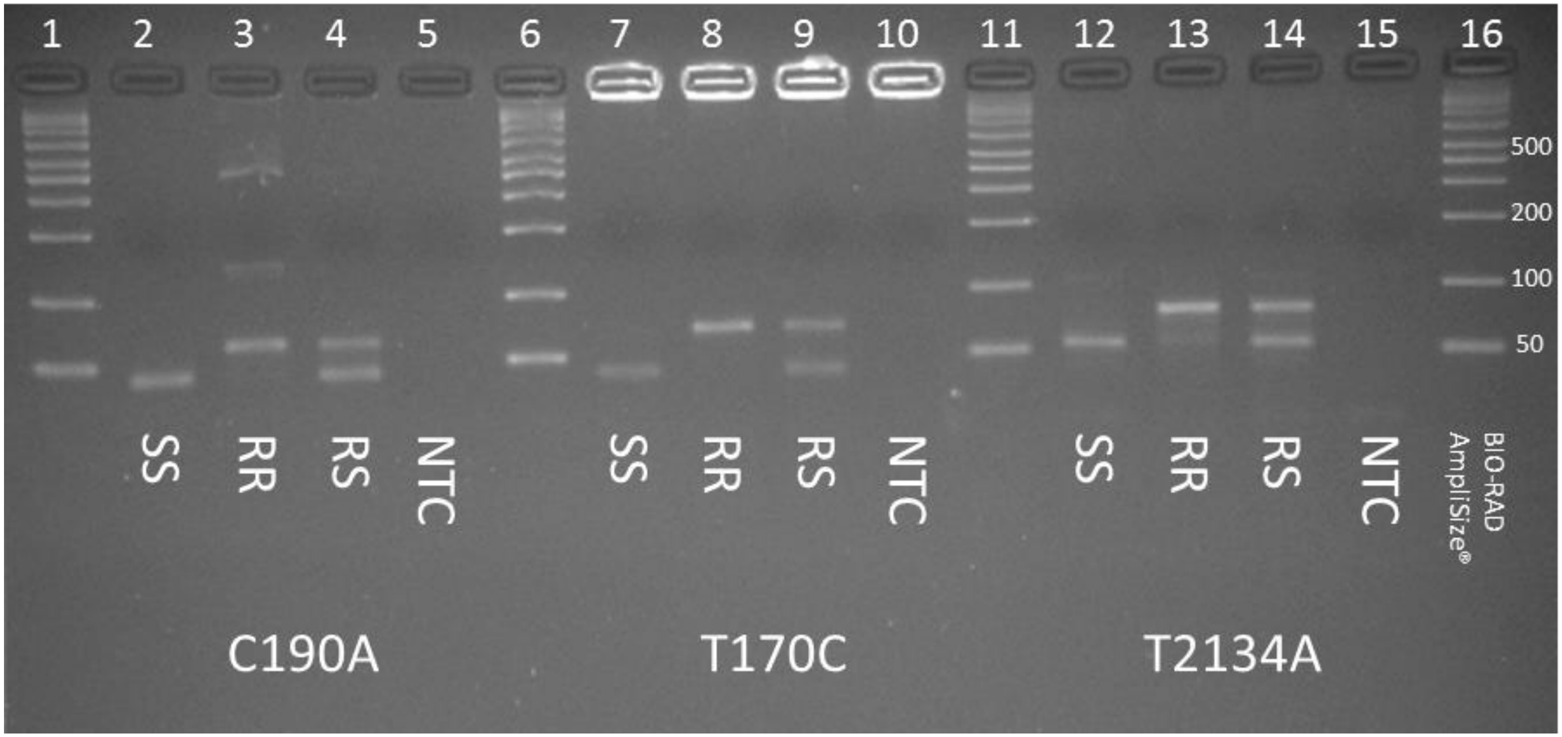

1. Birdsell DN, Pearson T, Price EP, Hornstra HM, Nera RD, Stone N, Gruendike J, Kaufman EL, Pettus AH, Hurbon AN *et al*: **Melt analysis of mismatch amplification mutation assays (Melt-MAMA): a functional study of a cost-effective SNP genotyping assay in bacterial models**. *PloS one* 2012, **7**(3):e32866.
