# Supplementary material for: Multiple mutations in the para-sodium channel gene are associated with pyrethroid resistance in Rhipicephalus microplus from the United States and Mexico
Source: Parasit Vectors. 2014 Oct 1;7:456. doi: 10.1186/s13071-014-0456-z (PMC4189684; doi:10.1186/s13071-014-0456-z)
Supplement: Additional file 5: Figure S2. — Nucleotide sequence alignment for the exon encoding domain II from Rhipicephalus microplus mRNA sequence (putative sodium channel accession number: [GenBank:AF134216.2]) with two resistance alleles and three additional fly species that are known to harbour kdr and super-kdr mutations. [file 13071_2014_456_MOESM5_ESM.pdf]

Additional file 4: Figure S2. Nucleotide sequence alignment for the exon encoding domain II from *Rhipicephalus microplus* mRNA sequence (putative sodium channel accession number [GenBank:AF134216.2]) with two resistance alleles and three additional fly species that are known to harbour *kdr* and *super-kdr* mutations. Sequences for *R. microplus* resistance alleles were generated by cloning and sequencing nine individual ticks that were heterozygous at both the T170C and C190A SNP locations (MLG 2 from Table 2). The *R. microplus* T170C (putative *super-kdr*) and C190A (*kdr*) SNPs are boxed; note that the T170C SNP occurs at the same position known to correlate with *super-kdr* in the house fly (*Musca domestica*) and horn fly (*Haemotobia irritans*), and the C190A SNP shares the same state associated with knockdown resistance (*kdr*) in the white fly (*Bemisia tabaci*). Nucleotide positions follow that of *R. microplus* accession number [GenBank:AF134216.2], while the vertical numbers represents nucleotide positions for each individual accession. The asterisks represent resistant sequences in the three fly species. Mutant nucleotide positions are based on previous publications from Williamson et al. [1], Guerrero et al. [2], and Morin et al. [3]. Accession numbers are as follows: *R. microplus* resistant [GenBank:KM073928 and KM073929], *M. domestica* [GenBank:U38814], *H. irritans* [GenBank:U83872], and *B. tabaci* [GenBank:AJ440727.1].

```
                                       10        20        30        40        50        60        70
                              ....|....|....|....|....|....|....|....|....|....|....|....|....|....|
R. microplus-AF134216.2       TACTTCCGCGAAGGCTGGAATATCTTCGATTTCCTCATCGTCGCGCTCTCCTTAATCGAACTAAGTTTGG 70
R. microplus resistant-T170C  ---------------------------------------------------------------------- 70
R. microplus resistant-C190A  ---------------------------------------------------------------------- 70
M. domestica-U38814           .......AG...........C..T.........A.T..T..G..CT.G..TC.GC.G...T.GG.CC... 2653
M. domestica-U38814*          .......AG...........C..T.........A.T..T..G..CT.G..TC.GC.G...T.GG.CC... 2653
H. irritans-U83872            ---------..G...........T.........A.T.....T..CT.G..T...C.G...T.GG.CC... 61
H. irritans-U83872*           ---------..G...........T.........A.T.....T..CT.G..T...C.G...T.GG.CC... 61
B. tabaci-AJ440727.1          ----------------------------------------------------------------------
B. tabaci-AJ440727.1*         ----------------------------------------------------------------------

                                       80        90       100       110       120       130       140
                              ....|....|....|....|....|....|....|....|....|....|....|....|....|....|
R. microplus-AF134216.2       AAAACGTCCAAGGATTGTCTGTGCTACGTTCGTTTCGTCTGCTACGTGTGTTCAAGCTAGCCAAATCGTG 140
R. microplus resistant-T170C  ------------------------------------------............................ 140
R. microplus resistant-C190A  ------------------------------------------............................ 140
M. domestica-U38814           .GGGT.....G..CC....G...T.GA.AAGT......T....T.....A.....AT.G..A.....A.. 2723
M. domestica-U38814*          .GGGT.....G..CC....G...T.GA.AAGT......T....T.....A.....AT.G..A.....A.. 2723
H. irritans-U83872            ..GGT.....G..C.....C...T..A.AAGT......T....T.....A.....AT.G..A.....T.. 131
H. irritans-U83872*           ..GGT.....G..C.....C...T..A.AAGT......T....T.....A.....AT.G..A.....T.. 131
B. tabaci-AJ440727.1          -----------------------------------------------------------........C.. 11
B. tabaci-AJ440727.1*         -----------------------------------------------------------........C.. 11

                                      150       160       170       180       190       200       210
                              ....|....|....|....|....|....|....|....|....|....|....|....|....|....|
R. microplus-AF134216.2       GCCTACCCTTAACCTGCTCATCTCTATCATGGGGAAAACCATCGGTGCCCTCGGGAACTTGACCTTTGTC 210
R. microplus resistant-T170C  .............................C........................................ 210
R. microplus resistant-C190A  .................................................A.................... 210
M. domestica-U38814           ...C..A..G..TT.A.....T..G..T.....CCGG..A..G.....AT.G..T..TC....A.....A 2793
M. domestica-U38814*          ...C..A..G..TT.A.....T..G..T.C...CCGG..A..G.....AT.G..T..TC....A.....A 2793
H. irritans-U83872            ...C.....G...T.A.....T..A..T.....CCGG..A..G.....AT.G..T..TC.A..A.....A 201
H. irritans-U83872*           ...C.....G...T.A.....T..A..T.C...CCGG..A..G.....AT.G..T..TC.A..A.....A 201
B. tabaci-AJ440727.1          ...A..TT.G..T...T.G..T..A........CCG...AG.T..G...T.A..A..T.....T.....T 81
B. tabaci-AJ440727.1*         ...A..TT.G..T...T.G..T..A........CCG...AG.T..G...A.A..A..T.....T.....T 81

                                      220       230       240       250       260       270
                              ....|....|....|....|....|....|....|....|....|....|....|....|....|....
R. microplus-AF134216.2       CTGGGAATCATCATCTTCATCTTCGCCGTGATGGGAATGCAACTCTTTGGCAAGAACTACGAAGAAAGT 279
R. microplus resistant-T170C  ..................................................................... 279
R. microplus resistant-C190A  ..................................................................... 279
M. domestica-U38814           ..TT.C..T..............T....................T..C..A........TATT..CCAC 2862
M. domestica-U38814*          ..TT.C..T..............T....................T..C..A........TATT..CCAC 2862
H. irritans-U83872            ..TT.C..T..............T.....T..............T..C..A........TATT..TCAC 270
H. irritans-U83872*           ..TT.C..T..............T.....T..............T..C..A........TATT..TCAC 270
B. tabaci-AJ440727.1          T..T.T.....T..T.....T..T..T.................A..C..G.....T..TAC...C.A. 150
B. tabaci-AJ440727.1*         T..T.T.....T..T.....T..T..T.................A..C..G.....T..TAC...C.A. 150
```

1. Williamson MS, Martinez-Torres D, Hick CA, Devonshire AL: **Identification of mutations in the housefly *para*-type sodium channel gene associated with knockdown resistance (*kdr*) to pyrethroid insecticides**. *Mol Gen Genet* 1996, **252**(1-2):51-60.
2. Guerrero FD, Jamroz RC, Kammlah D, Kunz SE: **Toxicological and molecular characterization of pyrethroid-resistant horn flies, *Haematobia irritans*: identification of *kdr* and *super-kdr* point mutations**. *Insect Biochem Molec* 1997, **27**(8-9):745-755.
3. Morin S, Williamson MS, Goodson SJ, Brown JK, Tabashnik BE, Dennehy TJ: **Mutations in the *Bemisia tabaci para* sodium channel gene associated with resistance to a pyrethroid plus organophosphate mixture**. *Insect Biochem Molec* 2002, **32**(12):1781-1791.
