# Supplementary material for: Multiple mutations in the para-sodium channel gene are associated with pyrethroid resistance in Rhipicephalus microplus from the United States and Mexico
Source: Parasit Vectors. 2014 Oct 1;7:456. doi: 10.1186/s13071-014-0456-z (PMC4189684; doi:10.1186/s13071-014-0456-z)
Supplement: Additional file 6: Figure S3. — Amino acid sequence alignment for domain II from Rhipicephalus microplus (putative sodium channel accession number: AAD23600.2) with two resistance alleles and three additional fly species that are known to harbour L925I (kdr) and M918T (super-kdr) substitutions. [file 13071_2014_456_MOESM6_ESM.pdf]

Additional file 5: Figure S3. Amino acid sequence alignment for domain II from *Rhipicephalus microplus* (putative sodium channel accession number: AAD23600.2) with two resistance alleles and three additional fly species that are known to harbour L925I (*kdr*) and M918T (*super-kdr*) substitutions. Amino acid sequences for *R. microplus* resistance alleles were translated from the nucleotide sequences shown in Additional file 4: Figure S2. The *R. microplus* methionine to threonine (putative *super-kdr*) and leucine to isoleucine (*kdr*) substitutions are boxed and show the precise alignment of the putative *super-kdr* substitution to M918T, which is known to correlate with *super-kdr* in two fly species (*Musca domestica* and *Haemotobia irritans*) and the *kdr* substitution to T925I in the white fly (*Bemisia tabaci*). The horizontal ruler represents amino acid positions for *R. microplus* accession number [GenBank:AAD23600.2], while the vertical numbers represents amino acid positions for each individual species according to their respective accession numbers. Amino acid substitution locations are based on previous publications from Williamson et al. [1], Guerrero et al. [2], and Morin et al. [3]. Accession numbers are as follows: *M. domestica* [GenBank:AAB47605.1], *H. irritans* [GenBank:AAC12794.1], and *B. tabaci* [GenBank:CAD29437.1].

```
                                            10        20        30        40        50        60        70        80        90
                                    ....|....|....|....|....|....|....|....|....|....|....|....|....|....|....|....|....|....|...
R. microplus-AAD23600.2             YFREGWNIFDFLIVALSLIELSLENVQGLSVLRSFRLLRVFKLAKSWPTLNLLISIMGKTIGALGNLTFVLGIIIFIFAVMGMQLFGKNYEES 93
R. microplus resistant-M918T        --------------------------------------..................T.................................... 93
R. microplus resistant-L925I        --------------------------------------.........................I............................. 93
M. domestica-AAB47605.1             ..Q........I......L..G..G.................................R.M..........C..................IDH 954
M. domestica-AAB47605.1-M918T       ..Q........I......L..G..G...............................T.R.M..........C..................IDH 954
H. irritans-AAC12794.1              ---........I......L..G..G.................................R.M..........C..................IDH 90
H. irritans-AAC12794.1-M918T        ---........I......L..G..G...............................T.R.M..........C..................IDH 90
B. tabaci-CAD29437.1                -------------------------------------------...............R.V..........C..................TDN 50
B. tabaci-CAD29437.1-L925I          -------------------------------------------...............R.V..I.......C..................TDN 50
```


1. Williamson MS, Martinez-Torres D, Hick CA, Devonshire AL: **Identification of mutations in the housefly *para*-type sodium channel gene associated with knockdown resistance (*kdr*) to pyrethroid insecticides**. *Mol Gen Genet* 1996, **252**(1-2):51-60.
2. Guerrero FD, Jamroz RC, Kammlah D, Kunz SE: **Toxicological and molecular characterization of pyrethroid-resistant horn flies, *Haematobia irritans*: identification of *kdr* and *super-kdr* point mutations**. *Insect Biochem Molec* 1997, **27**(8-9):745-755.
3. Morin S, Williamson MS, Goodson SJ, Brown JK, Tabashnik BE, Dennehy TJ: **Mutations in the *Bemisia tabaci para* sodium channel gene associated with resistance to a pyrethroid plus organophosphate mixture.** *Insect Biochem Molec* 2002, **32**(12):1781-1791.
